# Quorum Sensing Controls Adaptive Immunity through the Regulation of Multiple CRISPR-Cas Systems

**DOI:** 10.1016/j.molcel.2016.11.012

**Published:** 2016-12-15

**Authors:** Adrian G. Patterson, Simon A. Jackson, Corinda Taylor, Gary B. Evans, George P.C. Salmond, Rita Przybilski, Raymond H.J. Staals, Peter C. Fineran

**Affiliations:** 1 Department of Microbiology and Immunology, University of Otago, P.O. Box 56, Dunedin 9054, New Zealand; 2 Ferrier Research Institute, Victoria University of Wellington, 69 Gracefield Road, Lower Hutt 5010, New Zealand; 3 Department of Biochemistry, University of Cambridge, Tennis Court Road, Cambridge CB2 1QW, UK

**Keywords:** CRISPR-Cas, regulation, quorum sensing, bacterial communication, horizontal gene transfer, phage resistance

## Abstract

Bacteria commonly exist in high cell density populations, making them prone to viral predation and horizontal gene transfer (HGT) through transformation and conjugation. To combat these invaders, bacteria possess an arsenal of defenses, such as CRISPR-Cas adaptive immunity. Many bacterial populations coordinate their behavior as cell density increases, using quorum sensing (QS) signaling. In this study, we demonstrate that QS regulation results in increased expression of the type I-E, I-F, and III-A CRISPR-Cas systems in *Serratia* cells in high-density populations. Strains unable to communicate via QS were less effective at defending against invaders targeted by any of the three CRISPR-Cas systems. Additionally, the acquisition of immunity by the type I-E and I-F systems was impaired in the absence of QS signaling. We propose that bacteria can use chemical communication to modulate the balance between community-level defense requirements in high cell density populations and host fitness costs of basal CRISPR-Cas activity.

## Highlights

• Quorum sensing regulates the type I-E, I-F, and III-A CRISPR-Cas systems in *Serratia*
• SmaR represses *cas* gene and CRISPR expression in the absence of AHL signals
• Both interference and adaptation are modulated by quorum sensing
• Bacteria coordinate their defenses based on cell density and the risk of infection

## Graphical Abstract

**Figure:**
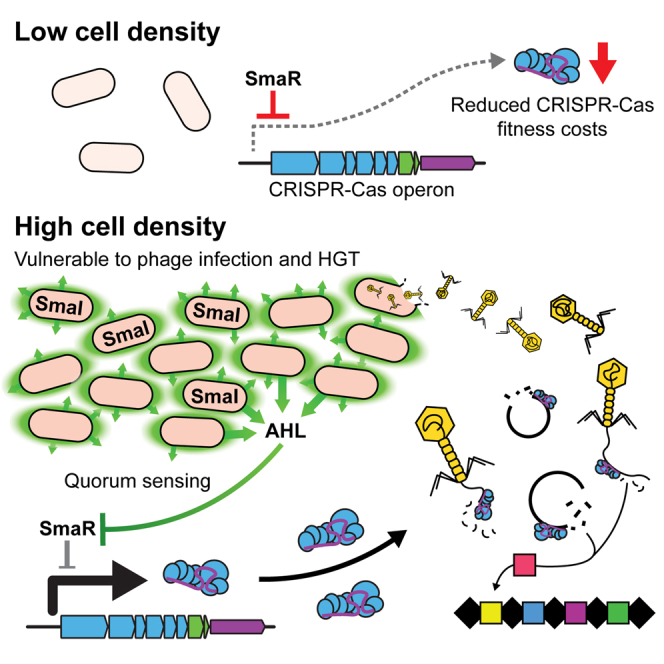

## Teaser

Patterson et al. examined quorum sensing and the function of three CRISPR-Cas systems in *Serratia*. They discovered that bacteria can use chemical communication to coordinate CRISPR-Cas immune defenses at high cell densities.

## Introduction

In nature, bacteria persist in myriad environments, from sparse populations to localized communities of high cell density, including cell chains, microcolonies, and biofilms (Hall-Stoodley et al., 2004). These bacterial populations can provide collective advantages, but a trade-off may be an increased susceptibility to bacteriophage (phage) infection (Abedon, 2012) and invasion by mobile genetic elements (Babic et al., 2011, Fuqua and Winans, 1994, Pinedo and Smets, 2005). Thus, it has been theorized that formation of microbial groups is only advantageous in times of low phage abundance, or if the threat is attenuated through elevated bacterial defenses (Abedon, 2012). It is well established that groups of bacteria regulate their behavior in response to cell density through QS, which is a widespread form of population-level communication (Miller and Bassler, 2001). As cell density increases, QS mediates accumulation of extracellular chemical signals, which are sensed by nearby bacteria, resulting in altered gene expression (Miller and Bassler, 2001).

In response to viral invasion and potentially deleterious impacts of HGT, bacteria possess an arsenal of defense systems (Dy et al., 2014, Wright et al., 2016). The CRISPR-Cas (clustered regularly interspaced short palindromic repeats [CRISPR] and their CRISPR-associated [Cas] proteins) systems provide adaptive sequence-specific immunity against foreign elements, such as phages and plasmids (Barrangou et al., 2007, Marraffini and Sontheimer, 2008). Immunity is first generated during adaptation when short invader-derived sequences (spacers) are integrated into CRISPR arrays (Amitai and Sorek, 2016, Wright et al., 2016). Second, the CRISPR arrays are transcribed and processed by Cas proteins, and in some cases host proteins, into short non-coding CRISPR RNAs (crRNAs). Finally, the crRNAs are assembled with Cas proteins into complexes that identify complementary invading nucleic acids and mediate their destruction—a process termed interference. The evolutionary success of CRISPR-Cas systems is evident from their broad distribution within bacteria and archaea (Makarova et al., 2015). However, although CRISPR-Cas systems confer tangible benefits, there are associated fitness costs (Vale et al., 2015, Westra et al., 2015). Hence, multiple systems are transcriptionally regulated (Arslan et al., 2013), which might enable physiological responsiveness to a changing environment and, thereby, a net cost-benefit balance. Since bacterial defensive requirements are predicted to change relative to population density (Abedon, 2012), we hypothesized that CRISPR-Cas immunity could be integrated into the host QS circuit, allowing increased defense at higher cell densities.

## Results

### Expression of Three CRISPR-Cas Systems Is QS Dependent

**Figure 1:**
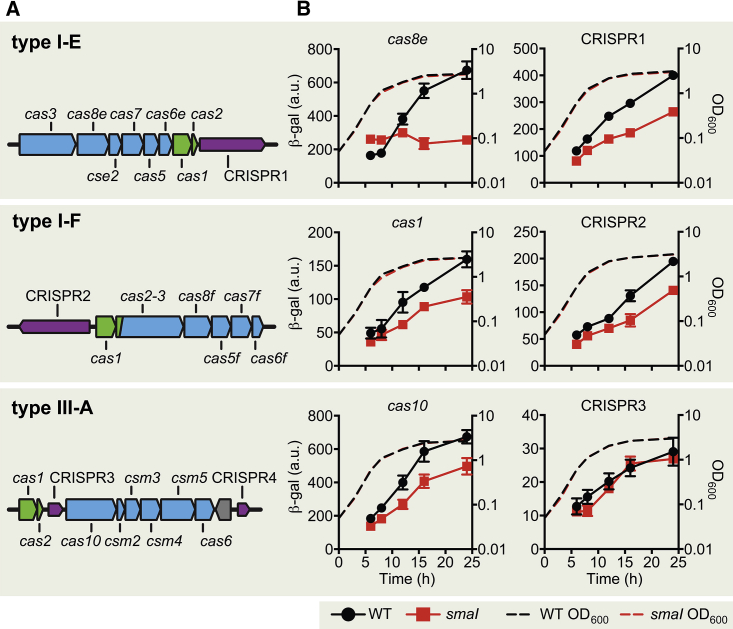
Quorum Sensing Regulates Expression of Three Distinct CRISPR-Cas Systems (A) Schematic of the *Serratia* sp. ATCC39006 CRISPR-Cas systems. Genes encoding interference or adaptation machinery are colored blue or green, respectively. The four CRISPR arrays—CRISPR1 (I-E, 52 spacers), CRISPR2 (I-F, 57 spacers), CRISPR3 (III-A, 9 spacers), and CRISPR4 (III-A, 8 spacers)—are colored purple. (B) *cas*::*lacZ* and CRISPR::*lacZ* activity and growth for each of the type I-E, type I-F, and type III-A reporter strains in the WT and *smaI* mutant backgrounds (Table S1). Differences in activity between WT and *smaI* beyond 12 hr were statistically significant (p ≤ 0.05) for each reporter except CRISPR3 (two-way analysis of variance [ANOVA] with Bonferroni’s multiple comparisons test). Data shown are the mean ± SD (n = 3). Figure S1 contains data for *smaI*::*lacZ* expression and C4-HSL production in addition to type I-E *cas3* and type III-A *cas1* and CRISPR4::*lacZ* expression. Complementation of all CRISPR-Cas reporters with C4-HSL is shown in Figure S1.

To test the role of QS in CRISPR-Cas regulation, we used *Serratia* sp. ATCC39006, which possesses a LuxIR-type QS system (Thomson et al., 2000) and three CRISPR-Cas systems (type I-E, I-F, and III-A), each with at least one CRISPR array (Figure 1A). Quorum sensing in Gram-negative bacteria typically utilizes LuxI family proteins to generate *N*-acyl homoserine lactone (AHL) signals, which are sensed by LuxR-type transcriptional regulators (Miller and Bassler, 2001). In *Serratia*, the *luxIR* homologs, *smaI* and *smaR*, control secondary metabolite production and motility, and SmaI produces predominantly *N*-butanoyl-l-homoserine lactone (C4-HSL) (Fineran et al., 2005, Thomson et al., 2000). Under our experimental conditions, the transcription of both *smaI* and AHL levels rose as cell densities increased, peaking at late exponential growth as cultures transitioned into stationary phase (Figure S1). To examine the effects of QS on CRISPR-Cas, we assessed *cas* operon and CRISPR expression in the wild-type (WT) and a signal-deficient *smaI* mutant throughout growth (Figures 1B and S1). Remarkably, expression of *cas* operons for all three CRISPR-Cas systems, as well as CRISPR1 (type I-E) and CRISPR2 (type I-F), was significantly reduced in the absence of AHL signal production (Figure 1B). The CRISPR arrays associated with the type III-A system (CRISPR3 and CRISPR4) exhibited low expression in the WT and were not regulated by QS since no further reduction was detected in the *smaI* mutant (Figures 1B and S1). We were able to fully complement the *smaI* mutant throughout growth by the addition of chemically synthesized C4-HSL, thereby confirming that the decreased *cas* and CRISPR expression in the *smaI* mutant resulted from the lack of AHL production (Figure S1). In agreement with previous work examining QS controlled secondary metabolite production in *Serratia*, addition of C4-HSL did not induce precocious induction of gene expression in the WT (Slater et al., 2003). Overall, expression of one or both core components (*cas* genes or CRISPRs) from all three CRISPR-Cas systems was subject to QS control.

### CRISPR-Cas Regulation Involves the SmaR Repressor

**Figure 2:**
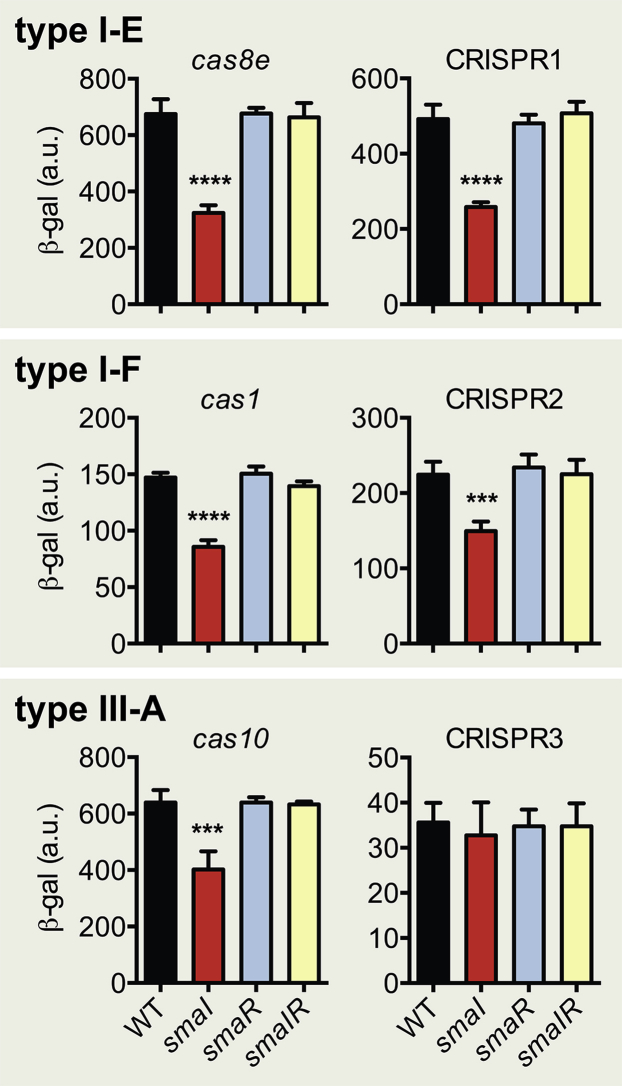
SmaR Represses CRISPR-Cas Expression in the Absence of QS Signals *cas*::*lacZ* and CRISPR::*lacZ* activity for each of the type I-E, I-F, and III-A reporter strains in the WT, *smaI* mutant, *smaR* mutant, and *smaIR* mutant backgrounds (Table S1) at 24 hr post inoculation. Statistical significance was calculated by one-way ANOVA with the Bonferroni’s multiple comparisons test (^∗∗∗^p ≤ 0.001, ^∗∗∗∗^p ≤ 0.0001). Data shown are the mean ± SD (n = 3). Expression of all reporters, including CRISPR4 activity, is shown throughout growth in Figure S2. Repression of the CRISPR-Cas reporters by expression of SmaR is shown in Figure S3.

In the absence of the AHLs, the SmaR transcriptional regulator acts as a DNA-binding repressor (Fineran et al., 2005, Slater et al., 2003, Thomson et al., 2000). At increased cell density, AHLs accumulate and bind SmaR, thereby inhibiting its DNA binding activity, resulting in elevated gene expression through a de-repression mechanism (Fineran et al., 2005). Mutation of *smaR* alone had no effect on *cas* and CRISPR expression throughout growth (Figures 2 and S2). The lack of enhanced expression in the *smaR* mutant is well established for genes previously shown to be controlled by QS in *Serratia* and is likely to be due to other required physiological and regulatory inputs (Fineran et al., 2005). Deletion of *smaR* in the *smaI* mutant restored expression of the *cas* operons and CRISPR arrays throughout growth (Figures 2 and S2), demonstrating that, in the absence of AHL production, SmaR acts as a repressor of CRISPR and *cas* gene expression. In agreement, plasmid-encoded SmaR caused significantly reduced expression from each of the QS-regulated CRISPR and *cas* promoters but not from a non-QS regulated control promoter (Figure S3). The SmaR-mediated repression observed using this system was similar to the reduction in CRISPR and *cas* expression upon deletion of *smaI* in *Serratia*. Therefore, these results demonstrate that SmaR represses CRISPR-Cas expression in the absence of the QS signaling molecules.

### Quorum Sensing Modulates CRISPR Interference

**Figure 3:**
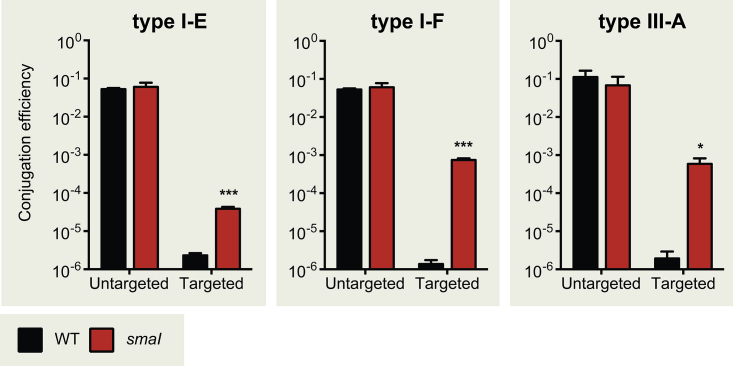
Quorum Sensing Is Required for Heightened CRISPR-Cas Interference Conjugation efficiency of untargeted plasmids or plasmids targeted by the type I-E, type I-F, or type III-A systems in the WT or *smaI* mutant backgrounds. Conjugation efficiency was scored as transconjugants/recipients. Statistical significance was assessed by unpaired two-tailed t test (^∗^p ≤ 0.05, ^∗∗∗^p ≤ 0.001). Data shown are the mean ± SD (n = 3). Complementation of all phenotypes using C4-HSL is shown in Figure S4.

Evidence that the CRISPR-Cas modules were regulated by the host QS circuit supported our hypothesis that defense against invaders would be elevated at high cell densities. To determine whether the transcriptional changes correlated with modulation of immunity, we exposed *Serratia* cells growing in high-density populations to donor bacteria that transfer, via conjugation, plasmids that mimicked invaders that were encountered previously. These plasmids contained sequences complementary to the first spacer present in CRISPR1, CRISPR2, or CRISPR3 for the type I-E, I-F, and III-A systems, respectively (Table S2). These target sequences are termed protospacers and, for the type I-E and I-F systems, included canonical protospacer adjacent motif (PAM) sequences that are necessary to evoke direct interference. In the WT populations, all three CRISPR-Cas systems were capable of robust interference of the respective target plasmids but not of untargeted control plasmids (Figure 3), demonstrating that each native system is functional. The conjugation efficiencies of untargeted (naive) control plasmids for the *smaI* mutant were comparable to the WT, demonstrating that there were no CRISPR-Cas-independent effects in this background. In contrast, the interference capability was significantly reduced in signaling-deficient populations (the *smaI* mutant) by ∼20-fold for type I-E, ∼500-fold for type I-F, and ∼240-fold for type III-A targeting (Figure 3). Unexpectedly, the type I-E system showed the weakest interference response to QS, despite having the strongest effect on the *cas8e* promoter (Figure 1). It is likely that the activity of other type I-E components might form a bottleneck for the overall level of interference, which is the case for *cas3* in the *E. coli* type I-E system (Majsec et al., 2016). The impaired interference in all three CRISPR-Cas systems could be rescued via the addition of exogenous QS signal (Figure S4). Despite the reduced levels of interference in the *smaI* mutant, we still observed relatively efficient recognition and destruction of the targeted plasmids by each of the CRISPR-Cas systems. Together, these results demonstrate that QS signaling modulates the efficiency of interference and is necessary to allow enhanced defense at high cell densities.

### Quorum Sensing Regulates Spacer Acquisition

**Figure 4:**
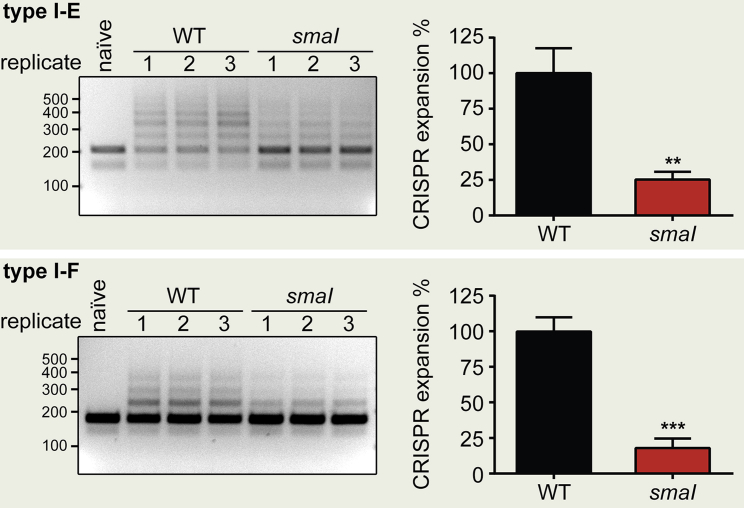
Quorum Sensing Boosts Adaptation in the Type I CRISPR-Cas Systems Spacer acquisition (CRISPR expansion) for the WT and *smaI* mutant strains was quantified after exposure to primed plasmids for either the type I-E or type I-F systems. The WT strain with the naive plasmid is also shown. CRISPR arrays were amplified by PCR and analyzed on 3% agarose gels. CRISPR expansion was normalized relative to the expansion observed in the WT (WT mean set as 100%). Statistical significance was assessed by unpaired two-tailed t test (^∗∗^p ≤ 0.01, ^∗∗∗^p ≤ 0.001). Data shown are the mean ± SD (n = 3). Complementation of all phenotypes using C4-HSL is shown in Figure S4.

Adaptation is a critical function of CRISPR-Cas systems, allowing generation of new immunity through spacer acquisition (Amitai and Sorek, 2016, Wright et al., 2016). Therefore, we asked whether this aspect of CRISPR-Cas was also regulated by QS. Two adaptation modes are known, naive and primed (Amitai and Sorek, 2016, Wright et al., 2016). During naive adaptation, spacers are acquired from elements to which no previous immunity exists, whereas primed adaptation, observed in type I systems, enhances acquisition of spacers from elements resembling those previously encountered (Datsenko et al., 2012). To examine adaptation in the WT and the *smaI* mutant, we tested their abilities to acquire spacers from either a “naive” plasmid, representing an unrecognized invader, or “primed” plasmids, representing escape mutants from targets of the type I-E or type I-F systems. Primed plasmids contained non-consensus PAMs to trigger primed acquisition of additional spacers (Table S2). For WT cells containing the naive plasmid, repeated passage to high cell density in the absence of antibiotic selection yielded no detectable naive spacer acquisition (Figure 4). In contrast, primed spacer acquisition from the escape plasmids was readily observed in the WT for both the type I-E and type I-F systems. Adaptation in the *smaI* mutant was reduced by ∼75% and ∼80% for the type I-E and type I-F systems, respectively (Figure 4). The impaired adaptation in both CRISPR-Cas systems was rescued via the addition of exogenous QS signal (Figure S4). In summary, QS-mediated elevation of CRISPR-Cas activity enhances the generation of immunological memory within high-density populations by promoting increased spacer acquisition.

## Discussion

Here we demonstrate that in a single strain, the expression of three different CRISPR-Cas systems (including types I and III) is regulated by the host QS circuit to significantly modulate immunity, including both interference and adaptation. The QS effect on adaptation is highly relevant with respect to bacterial population fitness because increased diversity of CRISPR spacers within communities restricts the success of phage escape mutants (van Houte et al., 2016). We show that QS-defective populations generate fewer new spacers during adaptation, hence less diversity, thereby highlighting the importance of cell-cell communication in stimulating population-level CRISPR-Cas resistance. This nascent immunity is further reinforced by the elevated interference invoked by spacers from the three different systems.

Our results demonstrate the importance of cell signaling in coordinating adaptive immunity when microbial groups are at high cell densities. Since HGT frequency, or phage spread, is less likely at low population densities (Abedon, 2012, Pinedo and Smets, 2005), defense does not need to be high, but it is still necessary. In agreement, we still observed efficient, albeit significantly reduced, CRISPR-Cas immunity under conditions mimicking low cell densities (i.e., the *smaI* mutant). Since SmaR is a repressor that is inactivated by AHLs, this particular QS system can be viewed as dampening down immunity at low cell density. The burden of CRISPR-Cas systems, such as lethal auto-immunity caused by self-targeting (Staals et al., 2016, Vercoe et al., 2013, Stern et al., 2010), might have provided selective pressure to evolve this “suppress when least required” mechanism. In contrast, successful phage infection of high cell density bacterial communities results in high localized viral loads that might overwhelm basal level CRISPR-Cas defenses. Thus, upregulation of CRISPR-Cas via QS facilitates transition to a heightened defensive state that is better suited to cope with high multiplicity of infection phage attacks.

As well as increasing general defense against invading elements, upregulation of CRISPR-Cas activity might also allow for an enhanced response to the stimulation of HGT or prophage release that can be triggered by QS. For example, diverse mobile elements coordinate their dissemination via QS, including AHL-based control of conjugative Ti plasmid transfer in *Agrobacterium tumefaciens* (Fuqua and Winans, 1994). Furthermore, many QS signals, including AHLs, *Pseudomonas* quinolone signal (PQS), and AI-2, can induce prophage induction in Gram-positive and Gram-negative bacteria (Fernández-Piñar et al., 2011, Hargreaves et al., 2014, Rossmann et al., 2015). It is salient that we observed QS-dependent regulation of type III-A activity. Type III are the only known CRISPR-Cas systems to target DNA in a transcription-dependent manner, which is thought to protect bacteria from active or induced prophages, while minimizing self-targeting of integrated prophages (Goldberg et al., 2014). Therefore, the QS-dependent response of the type III-A system might not only protect from phage infection, but also restrict the proliferation of viral progeny during prophage induction. Interestingly, a recent global metagenomic study highlighted the relevance of temperate phages in ecosystems with high bacterial abundances (Knowles et al., 2016), implying that the role of QS in prophage induction and CRISPR-Cas regulation could be ecologically significant.

The broad distribution of both CRISPR-Cas and QS systems within diverse bacteria suggests that QS-dependent regulation of immunity should be widespread. In support of this, we performed an analysis of published microarray data from *Pectobacterium atrosepticum* and discovered a significant reduction in type I-F *cas* gene expression in an AHL synthase mutant (*expI*) that was most pronounced at high cell density (Bowden et al., 2013). Likewise, in a *Burkholderia glumae* transcriptomic study, mutation of *luxI* homologs resulted in decreased expression of type I-F *cas* genes (Gao et al., 2015). Quorum sensing mechanisms used by bacteria are diverse, with peptide pheromones common in Gram-positives and the “universal” AI-2 signal produced by LuxS in many disparate bacteria. Furthermore, multiple CRISPR-Cas types (e.g., I-E, I-F, and III-A in *Serratia*) can be connected to QS circuits. Therefore, we predict that the control of adaptive immunity by QS is likely to be widespread across diverse bacteria and CRISPR-Cas types, irrespective of the precise signaling mechanism.

Fittingly, other phage defense systems operate under QS control (Høyland-Kroghsbo et al., 2013, Kolodkin-Gal et al., 2007). For example, an AHL-dependent reduction in receptors on *E. coli* limits infection by λ and χ phages (Høyland-Kroghsbo et al., 2013). Since *E. coli* does not produce AHLs, but has a LuxR sensor (SdiA), this might provide protection against broad host-range phages preying on neighboring bacteria. Within heterogeneous populations, analogous cross-species QS signaling could boost the CRISPR-Cas defenses of minority species, reducing the risk these individuals pose as vectors or reservoirs for phage spread. Other defense strategies provide population-level protection, such as abortive infection systems, which typically result in the “altruistic” suicide of infected cells. One such system, *mazEF* from *E. coli*, is regulated by a QS pentapeptide and limits phage P1 (Kolodkin-Gal et al., 2007). These suicidal defenses are most successful when bacteria are growing with spatial structure (Fukuyo et al., 2012). It is interesting that both abortive infection and CRISPR-Cas are most effective in populations at high cell density.

Because QS is an important regulator of CRISPR-Cas, invaders are likely to have evolved evasion mechanisms. In agreement, some phages encode acylases, enzymes that degrade AHLs, and others encode their own QS systems (Hargreaves et al., 2014). These phages might block or interfere with QS to improve their reproductive success in the face of CRISPR-Cas competition—akin to anti-CRISPR proteins (Bondy-Denomy et al., 2013). Indeed, phages have been engineered to express AHL-degrading enzymes that enhance their ability to disrupt biofilms by eliciting cell death and by inhibiting QS (Pei and Lamas-Samanamud, 2014). Our findings suggest that phage therapies that are combined with anti-QS strategies (e.g., engineered phages or anti-QS molecules) might assist in the evasion of CRISPR-Cas defense during treatment.

## Experimental Procedures

### Culture Conditions, Strains, and Plasmids

Tables S1 and S2 list all strains and plasmids used in this study, respectively, and Table S3 lists the oligonucleotides used. Details of strain and plasmid constructions are provided in the Supplemental Experimental Procedures. Unless otherwise stated, *Serratia* sp. ATCC39006 strains were grown at 30°C and *E. coli* strains at 37°C in Lysogeny Broth (LB), minimal medium agar (0.1% w/v (NH_4_)_2_SO_4_, 0.41 mM MgSO_4_, 0.2% w/v glucose, 40 mM K_2_HPO_4_, 14.7 mM KH_2_PO_4_ [pH 6.9–7.1], 1.5% w/v agar) or on LB-agar (LBA) plates containing 1.5% (w/v) agar. When required, media were supplemented with antibiotics as follows: ampicillin (Ap; 100 μg/mL), chloramphenicol (Cm; 25 μg/mL), kanamycin (Km; 50 μg/mL), spectinomycin (Sp; 50 μg/mL), and tetracycline (Tc; 10 μg/mL). 5-aminolevulinic acid (Ala; 50 μg/mL) was added for growth of ST18. Bacterial growth was measured in a Jenway 6300 Spectrophotometer at 600 nm (OD_600_), except when grown in 96-well microtiter plates, where it was measured in a Varioskan Flash Multimode Reader (Thermo Fisher Scientific) at 600 nm. All experiments were repeated in at least three biological replicates.

### AHL Production Assay

AHL production was assessed using bioassay plates as previously described (McClean et al., 1997). Briefly, the bioassay plates were prepared by seeding 100 mL of molten 0.75% LBA overlay with 1 mL of an ISTSO4 overnight culture, which was then poured over the surface of 1.5% LBA in a 25 cm × 25 cm square petri dish. Once set, holes were punched in the agar using a sterile cork borer. Samples for assay of AHL production were taken from cultures of WT (LacA) *Serratia* grown in multiple 1 mL aliquots of LB media in a 96 square deep-well plate (Labcon) incubated at 1,200 rpm at 30°C in a microplate shaker (BioProducts Incumix). Samples (1 mL) were pelleted by centrifugation at 13,000 rpm for 4 min, and the supernatants were sterilized using 0.22 μM syringe filters (Millipore). Supernatant samples for each time point were then used to fill the wells in the bioassay plate. The plate was subsequently incubated at 30°C for 24 hr, and the area of pigmentation surrounding each well was measured and reported as arbitrary units (a.u.). Supernatants from *smaI* mutant cultures were included at each time point and did not produce any detectable AHL.

### β-Galactosidase Expression Assays

Growth of bacterial strains containing the *lacZ* reporters and the β-galactosidase assays were performed as previously described (Patterson et al., 2015). The reporter strains contained a single chromosomal integration of the *lacZ* reporter fused to the ATG start codon of the various *cas* genes in the native genetic context, or to the start of the different CRISPR arrays. Therefore, they report on the expression from the various genes or arrays from their natural promoter positions within the chromosome (for a schematic see Figure S1 in Patterson et al., 2015). Briefly, bacteria were grown in 1 mL of LB with Tc in 96 square deep-well plates (Labcon) and incubated in a microplate shaker (BioProducts Incumix) at 1,200 rpm and 30°C. β-galactosidase assays were performed in a Varioskan Flash Multimode Reader (Thermo Fisher Scientific) as described previously using the fluorogenic substrate (4-Methylumbelliferyl β-D-galactoside; MUG) (Patterson et al., 2015, Ramsay, 2013). Relative fluorescent units (RFUs) per second were calculated using the linear increase in fluorescence, which was normalized to the OD_600_ of the sample (RFU/s/OD_600_). RFU/s/OD_600_ measurements are depicted as arbitrary units in the axis labels of relevant figures.

### Chemical Complementation Using *N*-Butanoyl-l-Homoserine Lactone

*N*-butanoyl-l-homoserine lactone (C4-HSL) was synthesized as described previously (Hodgkinson et al., 2011) and the chemical nature confirmed (Geske et al., 2005). C4-HSL was stored in dimethyl sulfoxide (DMSO) and was added at a final concentration of 0.5 μM at the start of growth in the chemical complementation experiments. In the control samples, an equivalent volume of DMSO was added as a solvent control. Growth and β-galactosidase assays were performed as described earlier. For complementation of interference and adaptation experiments, assays were performed as described later, but all plates and bacterial cultures were supplemented with 0.5 μM C4-HSL or DMSO (control).

### Conjugation Efficiency Assays

Conjugation efficiency was assessed in a similar manner to that described previously (Patterson et al., 2015, Richter et al., 2014). *E. coli* ST18 were used as donors for the conjugation of control (pPF719) and type I-E (pPF724) or type I-F (pPF722) targeted plasmids, or of control (pPF781) and type III-A (pPF1043) targeted plasmids. Plasmids pPF724, pPF722, and pPF1043 each contain a protospacer targeted by spacer 1 from either CRISPR1 (type I-E), CRISPR2 (type I-F), or CRISPR3 (type III-A), respectively. Recipient strains were WT (LacA) and *smaI* (LIS). Strains were grown overnight in LB with appropriate antibiotics, the OD_600_ adjusted to 1 and washed twice with LB. Donors and recipients were mixed in a 1:1 ratio, and 5 μL was spotted on 0.2 μm filters (Millipore) on LBA + Ala and incubated for 24 hr. Next, the filters were added to 2 mL PBS, the bacteria were resuspended, and dilution series were plated onto LB for recipient counts or with the addition of antibiotics for selection of transconjugant counts. For the type III-A experiments either 20 mM glucose or 0.02% arabinose was included in the plates for the filter matings or transconjugant selection, respectively. In all cases, conjugation efficiency was calculated as transconjugants per recipients.

### Adaptation Assays

Plasmids pPF719 (non-targeted “naïve” control), pPF1048 (“primed” type I-E), and pPF1032 (“primed” type I-F) were transferred from *E. coli* ST18 by conjugation into WT (LacA) and *smaI* (LIS) strains and plated on LBA + Tc. After PCR confirmation of the transconjugants, overnight cultures of each strain grown in the presence of Tc were used to inoculate fresh 5 mL cultures in LB without antibiotics in 20 mL universals. These were then incubated at 30°C with shaking and passaged for 6 days by daily transfer of 10 μL to 5 mL of fresh LB. CRISPR expansion (indicative of spacer acquisition) was determined by PCR directly on cells from passaged cultures (DreamTaq, Thermo Fisher Scientific) using primers PF1887 + PF1989 for CRISPR1 and PF1888 + PF1990 for CRISPR2. PCR products were separated by 3% agarose gel electrophoresis and stained with ethidium bromide, and spacer acquisition was quantified using ImageJ (Schneider et al., 2012).

## Author Contributions

Conceptualization, A.G.P., S.A.J., R.P., R.H.J.S., and P.C.F. Investigation, A.G.P., S.A.J., C.T., R.P., and R.H.J.S. Resources, G.B.E. and G.P.C.S. Formal Analysis, A.G.P., S.A.J., G.P.C.S., R.P., R.H.J.S., and P.C.F. Writing – Original Draft, A.G.P., S.A.J., R.H.J.S., and P.C.F. Writing – Review & Editing, A.G.P., S.A.J., G.B.E., G.P.C.S., R.P., R.H.J.S., and P.C.F. Funding Acquisition and Supervision, P.C.F.

## Accession Numbers

Unprocessed image files used to prepare the figures in this manuscript have been deposited in Mendeley Data and are available at http://dx.doi.org/10.17632/x55v9zf59x.1.

## Supplemental Information

Document S1. Supplemental Experimental Procedures, Figures S1–S4, and Tables S1–S3

Document S2. Article plus Supplemental Information
