# Supplementary material for: Quorum Sensing Controls Adaptive Immunity through the Regulation of Multiple CRISPR-Cas Systems
Source: Mol Cell. 2016 Dec 15;64(6):1102–8. doi: 10.1016/j.molcel.2016.11.012 (PMC5179492; doi:10.1016/j.molcel.2016.11.012)
Supplement: Document S1. Supplemental Experimental Procedures, Figures S1–S4, and Tables S1–S3 [file mmc1.pdf]

Molecular Cell, Volume *64*

Supplemental Information

Quorum Sensing Controls Adaptive Immunity through the Regulation of Multiple CRISPR-Cas Systems

Adrian G. Patterson, Simon A. Jackson, Corinda Taylor, Gary B. Evans, George P.C. Salmond, Rita Przybilski, Raymond H.J. Staals, and Peter C. Fineran

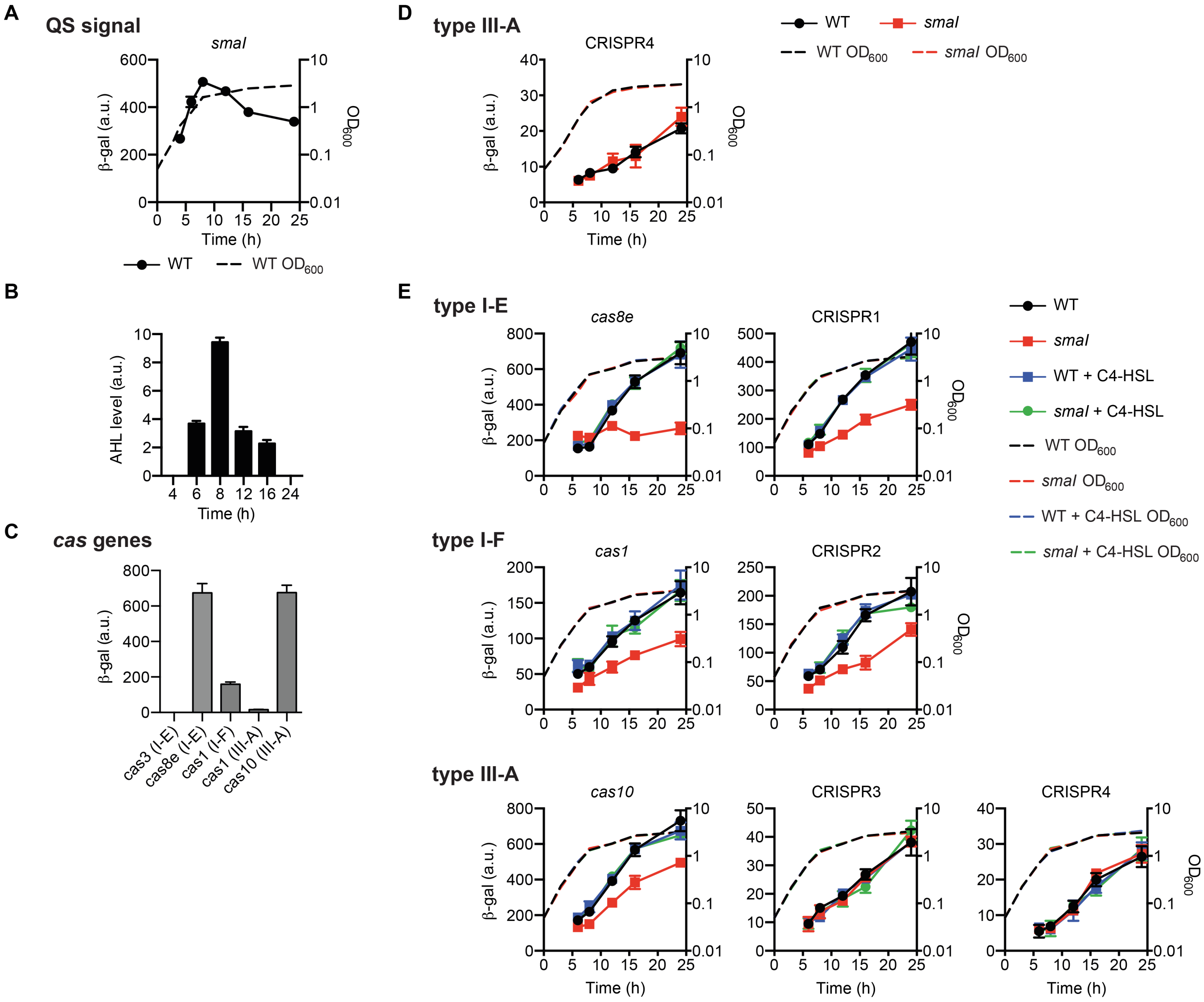

Figure S1. Production of C4-HSL signal via SmaI is responsible for upregulation of the I-E, I-F and III-A CRISPR-Cas systems at high cell density (related to Figure 1).

(A) Normalized *smaI*::*lacZ* reporter activity and cell growth of strain LC13. (B) AHL levels in the supernatant of WT cultures, measured using a bioassay. (C) Activity of the *cas*::*lacZ* reporters for the type I-E *cas3* (strain PCF209) and *cas8e* (strain PCF210), type I-F *cas1* (strain PCF211) and type III-A *cas1* (strain PCF212) and *cas10* (strain PCF213) promoters at 24 h post inoculation in the WT background. (D) Activity of CRISPR4::*lacZ* (solid lines) and growth (dashed lines) in the WT (strain PCF217) and *smaI* (strain PCF226) backgrounds. (E) Activity of *cas*::*lacZ* and CRISPR::*lacZ* for each of the type I-E, I-F and III-A CRISPR-Cas systems in the WT (strains PCF210, PCF211 and PCF213-PCF217) and *smaI* mutant backgrounds (PCF219, PCF220 and PCF222-PCF226) throughout growth ± 0.5 μM C4-HSL or DMSO (solvent control). Differences in activity between *smaI* + DMSO and *smaI* + C4-HSL beyond 12 h were statistically significant ($P \leq 0.05$) for each reporter except CRISPR3 and CRISPR4 (two-way analysis of variance (ANOVA) with the Bonferroni's multiple comparisons test). Data shown are the mean ± SD ($n = 3$).

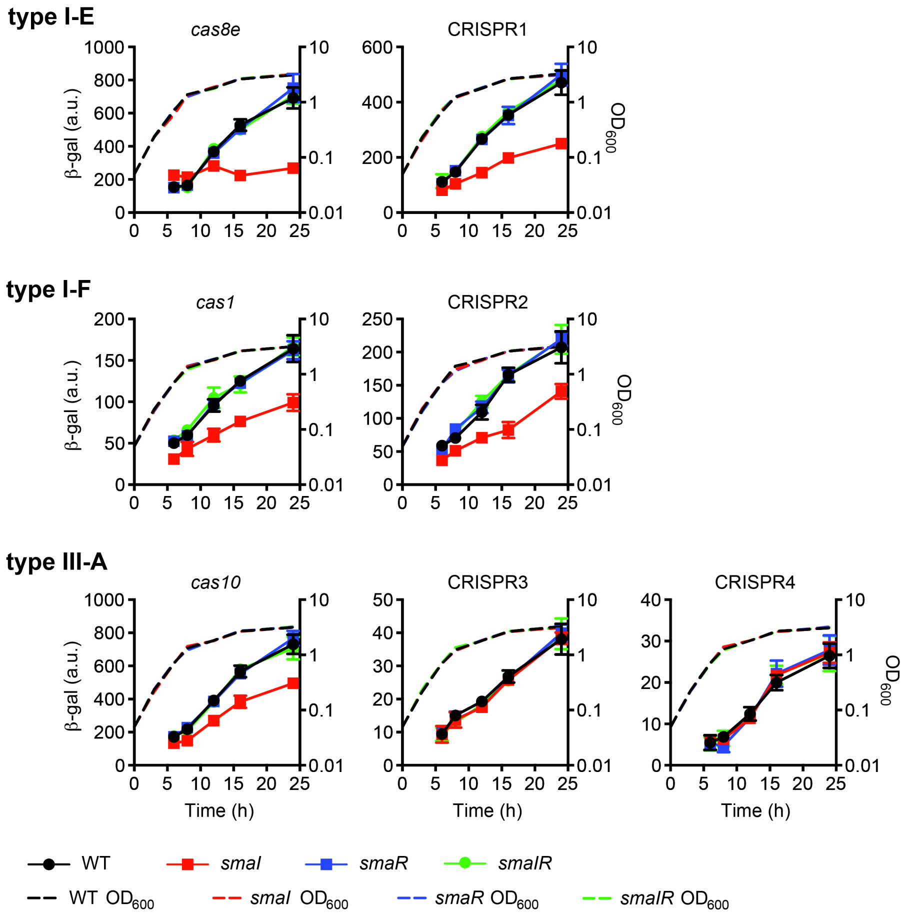

Figure S2. SmaR represses CRISPR-Cas expression in the absence of QS signals through growth (related to Figure 2). *cas*::*lacZ* and CRISPR::*lacZ* activity for each of the type I-E, type I-F and type III-A reporter strains in the WT (see Figure S1 for strain names), *smaI* mutant (see Figure S1 for strain names), *smaR* mutant (PCF228, PCF229 and PCF231-235) and *smaIR* mutant (PCF237, PCF238 and PCF240-244) backgrounds throughout growth. Differences in activity between *smaI* and all other strains beyond 12 h were statistically significant ($P \leq 0.05$) for each reporter except CRISPR3 and CRISPR4 (two-way analysis of variance (ANOVA) with the Bonferroni's multiple comparisons test). Data shown are the mean ± SD (n = 3).

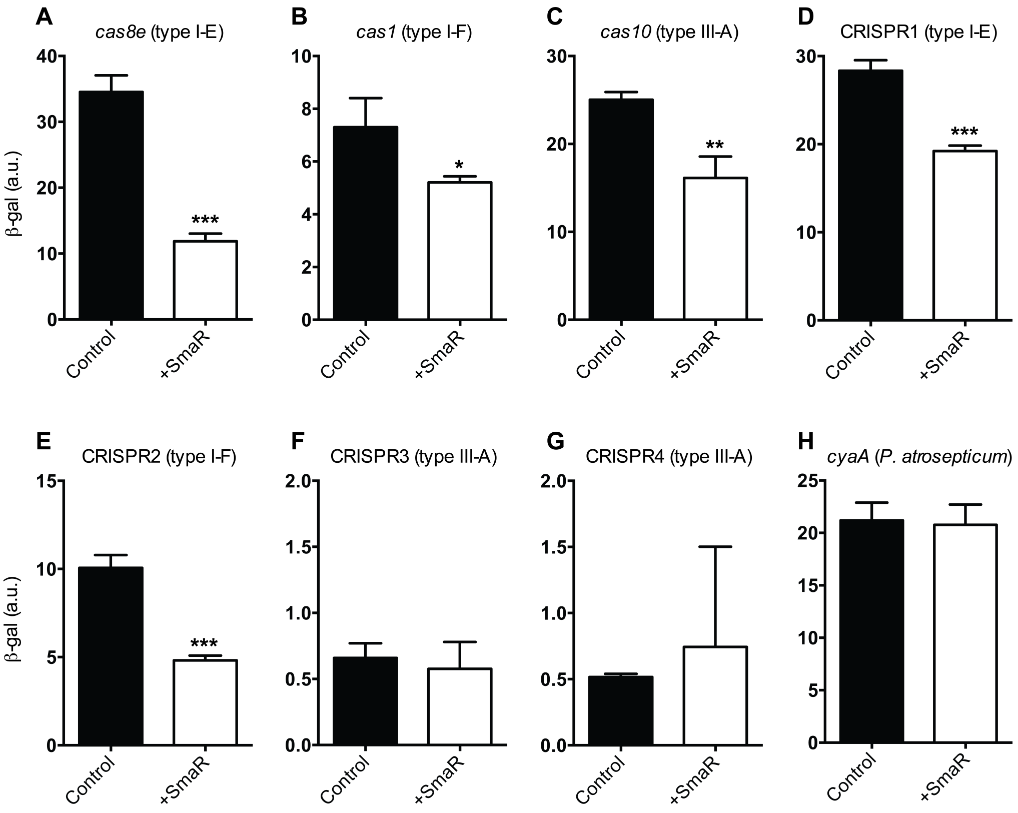

Figure S3. SmaR represses the type I-E, I-F and III-A CRISPR-Cas systems (related to Figure 2).

Normalized *cas*::*lacZ* activity in a heterologous system (*E. coli* CC118 λ*pir*) for each of the (A) type I-E *cas8e* (pPF714), (B) type I-F *cas1* (pPF715), (C) type III-A *cas10* (pPF717), (D) type I-E CRISPR1 (pPF861), (E) type I-F CRISPR2 (pPF862), (F) type III-A CRISPR3 (pPF863) and (G) type III-A CRISPR4 (pPF888) promoters ± SmaR (pPF972), at 3 h post induction with 0.1 mM IPTG. (H) Activity of a *P. atrosepticum cyaA*::*lacZ* (control) reporter in *E. coli* CC118 λ*pir* ± SmaR (pPF972), at 3 h post induction. Statistical significance was assessed by unpaired two-tailed t-test (* $P \leq 0.05$, ** $P \leq 0.01$, *** $P \leq 0.001$). Data shown are the mean ± SD ($n = 3$).

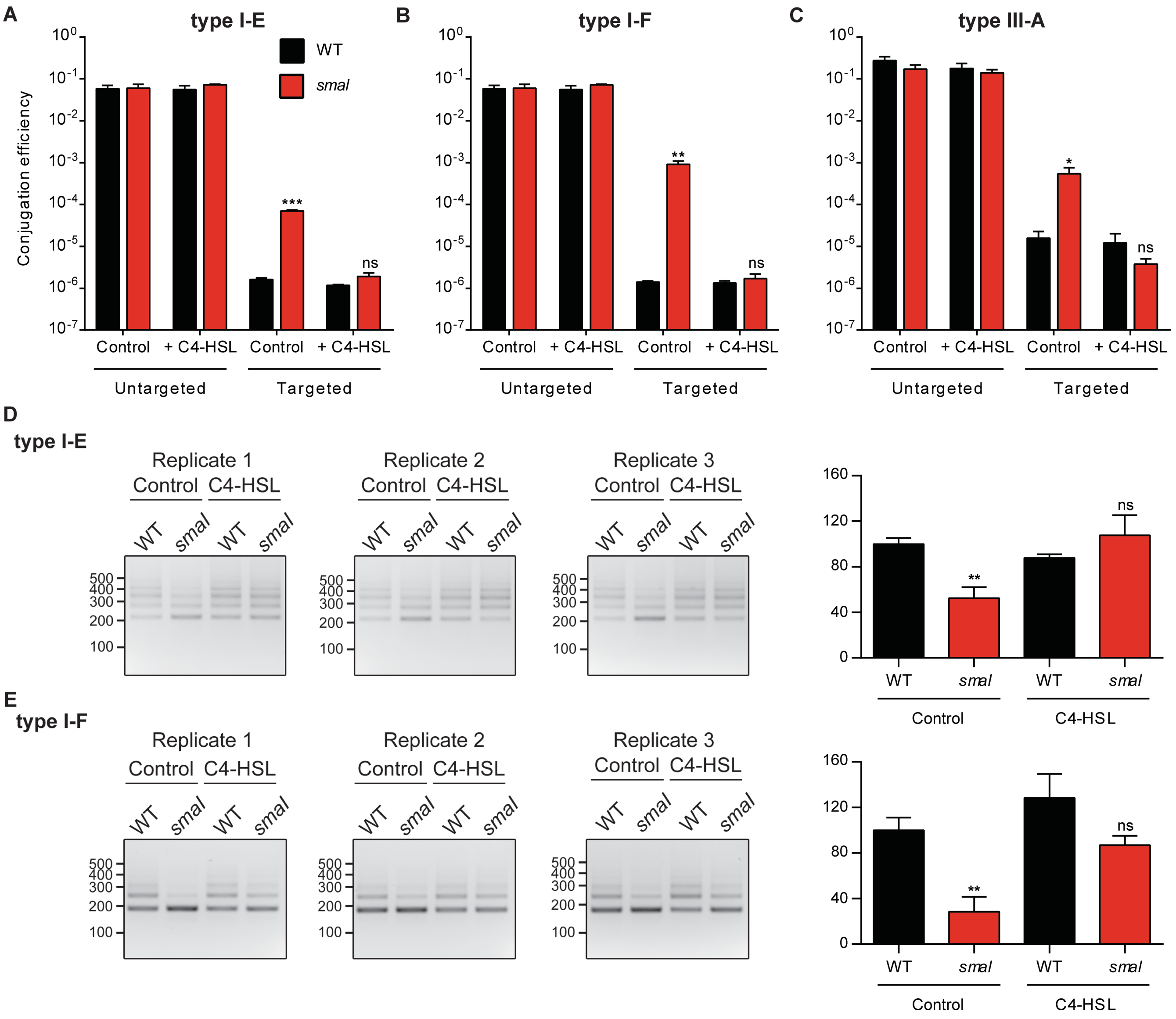

Figure S4. Addition of C4-HSL to the *smaI* mutant restores wild-type interference and adaptation for each CRISPR-Cas subtype (related to Figures 3 and 4).

Conjugation efficiency of untargeted control and targeted plasmids for the (A) type I-E (pPF719 control and pPF724 targeted), (B) type I-F (pPF719 control and pPF722 targeted) or (C) type III-A (pPF781 control and pPF1043 targeted) systems in WT (LacA) or *smaI* (LIS) mutant backgrounds. Conjugation efficiency was scored as transconjugants/recipients. Spacer acquisition (CRISPR expansion) for the WT or *smaI* mutant strains for either the (D) type I-E (pPF1048 primed) or (E) type I-F (pPF1032 primed) systems. CRISPR arrays were amplified by PCR and analyzed on 3% agarose gels. CRISPR expansion was normalized relative to the expansion observed in the WT control (WT control mean set as 100%). C4-HSL was added at a final concentration of 0.5 μM to the plates and bacterial cultures throughout the experiments and the controls included DMSO solvent. Statistical significance was assessed by unpaired t-test (* $P \leq 0.05$, ** $P \leq 0.01$, *** $P \leq 0.001$). Data shown are the mean ± SD ($n = 3$).

Table S1. Bacterial strains used in this study (related to Figures 1-4).

Name	Genotype/Phenotype	Reference
E. coli		
CC118 λ*pir*	*araD*, Δ(*ara*, *leu*), Δ*lacZ*74, *phoA*20, *galK*, *thi-1*, *rspE*, *rpoB*, *argE*, *recA*1, λpir	(Herrero et al., 1990)
DH5α	F$^-$, φ80Δd*lacZ*M15, Δ(*lacZYA–argF*)U169, *endA1*, *recA1*, *hsd*R17 ($r_K^- m_K^+$), *deoR*, *thi-1*, *sup*E44, λ$^-$, *gyr*A96, *rel*A1	Gibco/BRL
S17-1 λ*pir*	*recA, pro, hsdR, recA*::RP4-2-Tc::Mu, λ*pir,* Tmp^R, Sp^R, Sm^R	(de Lorenzo et al., 1990)
ST18	S17-1 λ*pir* Δ*hemA*	(Thoma and Schobert, 2009)
***Serratia* sp. ATCC 39006**		
LacA	lac- mutant generated by EMS mutagenesis, denoted “wild-type (WT)”	(Thomson et al., 2000)
ISTSO4	*smaI*::mini-Tn*5*Sm/Sp, *pigX*::mini-Tn*5lacZ1*, Sm^R/Sp^R, Km^R	(Fineran et al., 2005)
LC13	*smaI*::mini-Tn*5lacZ1*, Km^R	(Thomson et al., 2000)
LIS	*smaI*::mini-Tn*5*Sm/Sp, Sm^R/Sp^R	(Thomson et al., 2000)
MCR2000	*smaR*::*cat*, Cm^R	(Slater et al., 2003)
PCF208	*smaI*::mini-Tn*5*Sm/Sp, *smaR*::*cat*, Sm^R/Sp^R, Cm^R	This study
PCF209	WT type I-E *cas3*pro::*lacZ* (pPF713 integrant), Tc^R	This study
PCF210	WT type I-E *cas8e*pro::*lacZ* (pPF714 integrant), Tc^R	This study
PCF211	WT type I-F *cas1*pro::*lacZ* (pPF715 integrant), Tc^R	This study
PCF212	WT type III-A *cas1*pro::*lacZ* (pPF716 integrant), Tc^R	This study
PCF213	WT type III-A *cas10*pro::*lacZ* (pPF717 integrant), Tc^R	This study
PCF214	WT type I-E CRISPR1pro::*lacZ* (pPF861 integrant), Tc^R	This study
PCF215	WT type I-F CRISPR2pro::*lacZ* (pPF862 integrant), Tc^R	This study
PCF216	WT type III-A CRISPR3pro::*lacZ* (pPF863 integrant), Tc^R	This study
PCF217	WT type III-A CRISPR4pro::*lacZ* (pPF888 integrant), Tc^R	This study
PCF219	*smaI*::mini-Tn*5*Sm/Sp, type I-E *cas8e*pro::*lacZ* (pPF714 integrant), Tc^R, Sm^R/Sp^R	This study
PCF220	*smaI*::mini-Tn*5*Sm/Sp, type I-F *cas1*pro::*lacZ* (pPF715 integrant), Tc^R, Sm^R/Sp^R	This study
PCF222	*smaI*::mini-Tn*5*Sm/Sp, type III-A *cas10*pro::*lacZ* (pPF717 integrant), Tc^R, Sm^R/Sp^R	This study
PCF223	*smaI*::mini-Tn*5*Sm/Sp, type I-E CRISPR1pro::*lacZ* (pPF861 integrant), Tc^R, Sm^R/Sp^R	This study
PCF224	*smaI*::mini-Tn*5*Sm/Sp, type I-F CRISPR2pro::*lacZ* (pPF862 integrant), Tc^R, Sm^R/Sp^R	This study
PCF225	*smaI*::mini-Tn*5*Sm/Sp, type III-A CRISPR3pro::*lacZ* (pPF863 integrant), Tc^R, Sm^R/Sp^R	This study
PCF226	*smaI*::mini-Tn*5*Sm/Sp, type III-A CRISPR4pro::*lacZ* (pPF888 integrant), Tc^R, Sm^R/Sp^R	This study
PCF228	*smaR*::*cat*, type I-E *cas8e*pro::*lacZ* (pPF714 integrant), Tc^R, Cm^R	This study
PCF229	*smaR*::*cat*, type I-F *cas1*pro::*lacZ* (pPF715 integrant), Tc^R, Cm^R	This study
PCF231	*smaR*::*cat*, type III-A *cas10*pro::*lacZ* (pPF717 integrant), Tc^R, Cm^R	This study
PCF232	*smaR*::*cat*, type I-E CRISPR1pro::*lacZ* (pPF861 integrant), Tc^R, Cm^R	This study
PCF233	*smaR*::*cat*, type I-F CRISPR2pro::*lacZ* (pPF862 integrant), Tc^R, Cm^R	This study
PCF234	*smaR*::*cat*, type III-A CRISPR3pro::*lacZ* (pPF863 integrant), Tc^R, Cm^R	This study
PCF235	*smaR*::*cat*, type III-A CRISPR4pro::*lacZ* (pPF888 integrant), Tc^R, Cm^R	This study
PCF237	*smaI*::mini-Tn*5*Sm/Sp, *smaR*::*cat*, type I-E *cas8e*pro::*lacZ* (pPF714 integrant), Tc^R, Cm^R, Sm^R/Sp^R	This study
PCF238	*smaI*::mini-Tn*5*Sm/Sp, *smaR*::*cat*, type I-F *cas1*pro::*lacZ* (pPF715 integrant), Tc^R, Cm^R, Sm^R/Sp^R	This study
PCF240	*smaI*::mini-Tn*5*Sm/Sp, *smaR*::*cat*, type III-A *cas10*pro::*lacZ* (pPF717 integrant), Tc^R, Cm^R, Sm^R/Sp^R	This study
PCF241	*smaI*::mini-Tn*5*Sm/Sp, *smaR*::*cat*, type I-E CRISPR1pro::*lacZ* (pPF861 integrant), Tc^R, Cm^R, Sm^R/Sp^R	This study
PCF242	*smaI*::mini-Tn*5*Sm/Sp, *smaR*::*cat*, type I-F CRISPR2pro::*lacZ* (pPF862 integrant), Tc^R, Cm^R, Sm^R/Sp^R	This study
PCF243	*smaI*::mini-Tn*5*Sm/Sp, *smaR*::*cat*, type III-A CRISPR3pro::*lacZ* (pPF863 integrant), Tc^R, Cm^R, Sm^R/Sp^R	This study
PCF244	*smaI*::mini-Tn*5*Sm/Sp, *smaR*::*cat*, type III-A CRISPR4pro::*lacZ* (pPF888 integrant), Tc^R, Cm^R, Sm^R/Sp^R	This study

Table S2. Plasmids used in this study (related to Figures 1-4).

Name	Genotype/Phenotype	Reference
**Priming/interference**		
pBAD30	Arabinose inducible expression vector, Ap^R	(Guzman et al., 1995)
pQE-80LoriT-GFP	pQE-80LoriT derivative containing GFP, Ap^R	Josh Ramsay; unpublished
pQE-80LoriT-mCherry	pQE-80LoriT derivative containing mCherry, Ap^R	(Richter et al., 2014)
pPF719	Unprimed/naïve control pQE-80LoriT-GFP, Tc^R	This study
pPF1048	Primed type I-E CRISPR1 spacer 1 (TCA PAM) pQE-80LoriT-GFP-derivative, Tc^R	This study
pPF1032	Primed type I-F CRISPR2 spacer 2 containing a single nucleotide deletion at position 23 (GA PAM) pQE-80LoriT-GFP-derivative, Tc^R	This study
pPF724	Targeted type I-E CRISPR1 spacer 1 (CTT PAM) pQE-80LoriT-GFP-derivative, Tc^R	This study
pPF722	Targeted type I-F CRISPR2 spacer 1 (GG PAM) pQE-80LoriT-GFP-derivative, Tc^R	This study
pPF781	Untargeted/naïve control for the type III-A system, pBAD30 derivative, Cm^R	This study
pPF1043	Targeted type III-A pPF781 derivative containing a protospacer complementary to CRISPR3 spacer 1, Cm^R	This study
***Serratia* sp. ATCC 39006 *lacZ* reporters**		
pVIK107-Tc	pVIK107-derivative, integrative *lacZ* reporter plasmid, RP4 oriT, oriR6K, Tc^R	(Patterson et al., 2015)
pPF713	type I-E *cas3*pro::*lacZ* reporter, pVIK107-Tc-derivative, Tc^R	This study
pPF714	type I-E *cas8e*pro::*lacZ* reporter, pVIK107-Tc-derivative, Tc^R	This study
pPF715	type I-F *cas1*pro::*lacZ* reporter, pVIK107-Tc-derivative, Tc^R	This study
pPF716	type III-A *cas1*pro::*lacZ* reporter, pVIK107-Tc-derivative, Tc^R	This study
pPF717	type III-A *cas10*pro::*lacZ* reporter, pVIK107-Tc-derivative, Tc^R	This study
pPF861	type I-E CRISPR1pro::*lacZ* reporter, pVIK107-Tc-derivative, Tc^R	This study
pPF862	type I-F CRISPR2pro::*lacZ* reporter, pVIK107-Tc-derivative, Tc^R	This study
pPF863	type III-A CRISPR3pro::*lacZ* reporter, pVIK107-Tc-derivative, Tc^R	This study
pPF888	type III-A CRISPR4pro::*lacZ* reporter, pVIK107-Tc-derivative, Tc^R	This study
***Pectobacterium atrosepticum lacZ* reporters**		
pPF1008	*cyaA*::*lacZ* reporter, pVIK107-Tc-derivative, Tc^R	This study
**SmaR expression**		
pQE-80LoriT	pQE-80L (Qiagen) derivative containing RP4 oriT, Ap^R	(Richter et al., 2014)
pPF972	His_6-SmaR in pQE-80L-oriT, Ap^R	This study

Table S3. Oligonucleotides used in this study (related to Figures 1-4).

Name	Sequence (5'-3')	Notes	Restriction site(s)
PF209	TCGTCTTCACCTCGAGAAATC	F pQE-80L-oriT MCS	
PF210	GTCATTACTGGATCTATCAACAGG	R pQE-80L-oriT MCS	
PF565	CATAACACTGACAGAGGATCC	type I-E CRISPR1 sequencing	
PF575	GAACCCTTCAAAAATTGTCG	type I-F CRISPR2 sequencing	
PF610	CCTGGCAGTTCCCTACTCTC	R pBAD30 post-MCR	
PF633	GTGGATCTGGATGGACTGC	type I-E CRISPR1 sequencing	
PF1451	GCCACCTCGACCTGAAT	Targeted/primed construct sequencing	
PF1615	TTTTCCATGGGAATTGATTGGCTCCAATTC	F untargeted (no protospacer)	NcoI
PF1616	TTTTCCATGG**CN***CTGCAAAAATGCAGTAATATCAAGAAGTTTTAC* GAATTGATTGGCTCCAATTC	F type I-F CRISPR2 *protospacer* 1 (variable **PAM**)	NcoI
PF1617	TTTTCCATGG**ANG***TAAACAATTGCAGGACCAGCAAAATCTGTGG C*GAATTGATTGGCTCCAATTC	F type I-E CRISPR1 *protospacer* 1 (variable **PAM**)	NcoI
PF1623	TTTTCTAGAGGAATATAATTAACCTGATAATAAATG	F type I-E *cas3*pro	XbaI
PF1624	TTTCTGCAGCATCGCTTATTTTTAGAATAGAAC	R type I-E *cas3*pro	PstI
PF1625	TTTTCTAGATTTATCGGTGCGGGAAAGC	F type I-E *cas8e*pro	XbaI
PF1626	TTTCTGCAGCATGGATCTATCTCCTCAGTTA	R type I-E *cas8e*pro	PstI
PF1627	TTTTCTAGATCGTGTGCCAAACCCTTTTTTC	F type I-F *cas1*pro	XbaI
PF1628	TTTCTGCAGCATAATATATTCTCTGCTATCGCG	R type I-F *cas1*pro	PstI
PF1629	TTTTCTAGAATCCGCTCTCAGGGCTTTATG	F type III-A *cas1*pro	XbaI
PF1630	TTTCTGCAGCATGGTATCGATGTTCACTATGAAG	R type III-A *cas1*pro	PstI
PF1631	TTTTCTAGAAGTAGGTACTATTTCTTTGGTCC	F type III-A *cas10*pro	XbaI
PF1632	TTTCTGCAGCATTGACATCTCCTTGTGCC	R type III-A *cas10*pro	PstI
PF1642	CAGTCTAATTTGTTGACG	F screening type I-E *cas3*pro	
PF1643	GAGGTCGATATCTTTACC	F screening type I-E *cas8e*pro	
PF1644	CCATTTCTAAGCTGCCTG	F screening type I-F *cas1*pro	
PF1645	CCATGTTCTGATGCAGTC	F screening type III-A *cas1*pro	
PF1646	CATTACAGAATCATTGTCC	F screening type III-A *cas10*pro	
PF1666	CTTGACCCGGGCGCTAGCGGAGTGTATACTGGCTTACTATGTTG GCAC	F pBAD30 AraC	XmaI
PF1668	TTTTCTAGACTGGCAACAGATTGTGGAAC	F type I-E CRISPR1pro	XbaI
PF1669	TTTCTGCAGCATAGCTGTTTCCTTCTAAAAATATATACCTGTTTTA TAAGG	R type I-E CRISPR1pro	PstI
PF1670	TTTTCTAGAAAATCGTAGACTAATTATTTAATAGCG	F type I-F CRISPR2pro	XbaI
PF1671	TTTCTGCAGCATAGCTGTTTCCTAGTAACATTTCCACTTTAACGA TTTG	R type I-F CRISPR2pro	PstI
PF1672	TTTTCTAGACACACAACCGGCACACTC	F type III-A CRISPR3pro	XbaI
PF1673	TTTCTGCAGCATAGCTGTTTCCTAATGAAAATTTATAACCCATTG TTTTTATTTG	R type III-A CRISPR3pro	PstI
PF1674	TTTTCTAGACCCATTCAGGCACAGATTTTG	F type III-A CRISPR4pro	XbaI
PF1675	TTTCTGCAGCATAGCTGTTTCCTAATGAAAATTTATAACCCATTG TTTTTGTTTG	R type III-A CRISPR4pro	PstI
PF1698	CTCGGTACCATGGCATGCTGCAGTGGATGACCTTTTGAATGACC	F T4:Cm^R:T7 cassette	KpnI/NcoI/SphI /PstI
PF1699	GAGGGGTTTTTTGGGCCAGCCTCGCAGAGCAG	F OriT, overlaps PF1700	
PF1700	GAGGCTGGCCCAAAAAACCCCTCAAGACCCG	R T4:Cm^R:T7 cassette, overlaps PF1699	
PF1701	TTTGCCCGGGAGCGCTTTTCCGCTGC	R OriT	XmaI
PF1789	GATCCCGGGGTTTCAGGAAGGCGGCAAG	F primer *cyaA* pro	SmaI
PF1790	TTTTCTAGACAAGACGTTTCGCCTGATTTATC	R primer *cyaA* pro	XbaI
PF1834	GTTGGTGGTCGTGACGG	F screening type I-E CRISPR1pro and sequencing	
PF1835	GAATACGGCAGTGCTGGAG	F screening type I-F CRISPR2pro and sequencing	
PF1836	CCCAGAGGAAGACCGTC	F screening type III-A CRISPR3pro	
PF1837	CATCGTTCCATCATGCG	F screening type III-A CRISPR4pro	
PF1874	GGTCAATCAAGAATTTCACCAG	type I-E CRISPR1 sequencing	
PF1875	GGACAATACTTTTTGAGGATGAC	type I-E CRISPR1 sequencing	
PF1876	GTTTAGCTACTACTCCGCGCAGC	type I-F CRISPR2 sequencing	
PF1887	GTTAAGTCAGCAGGCGTTTAGTCG	R type I-E CRISPR1 spacer 2	

Table S3 (continued). Oligonucleotides used in this study (related to Figures 1-4).

Name	Sequence (5'-3')	Notes	Restriction site(s)
PF1888	CATCTGATGCTGACGACACTG	R type I-F CRISPR2 spacer 2	
PF1903	GTTGTTCCGCGAGACTATCGAC	type I-F CRISPR2 sequencing	
PF1904	CCAGGAATCACTATATCTGGCAAG	type I-F CRISPR2 sequencing	
PF1905	GCACTGAATGTTCGATATCATTAC	type I-E CRISPR1 sequencing	
PF1958	CTGGGATCCGTGTCTAATTCATTCTTTAAT	F *smaR*	BamHI
PF1959	GATCTGCAGTCATTCTGCGTCAGGGAG	R *smaR*	PstI
PF1989	TAAGTTAGTGTTCTTTAACAAGCAGGA	F type I-E CRISPR1 leader	
PF1990	CACGAAAATGATAATTGATGCTGAT	F type I-F CRISPR2 leader	
PF2034	TTTTCCATGG**N**C*TGGAAGCCGCCAGTGTCGTCAGATCAGATGT*G<br>AATTGATTGGCTCCAATTC	F type I-F CRISPR2 *protospacer* 2 containing a single nucleotide deletion at position 23 (variable **PAM**)	NcoI
PF2085	TTTTCCATGG**NSW***TAAACAATTGCAGGACCAGCAAAATCTGTGG*<br>*C*GAATTGATTGGCTCCAATTC	F type I-E CRISPR1 *protospacer* (variable **PAM**)	NcoI
PF2086	CTTGG*AAAAAAAACCGACACGTAGTGTGAAAGAAATTAGG*ATGAG<br>CATG	type III-A CRISPR3 *protospacer* 1, anneals to PF2087	KpnI/SphI compatible
PF2087	CTCAT*CCTAATTTCTTTCACACTACGTGTCGGTTTTTTTT*CCAAGG<br>TAC	type III-A CRISPR3 *protospacer* 1, anneals to PF2086	KpnI/SphI compatible

Note: In the oligonucleotide sequences, protospacers are in italics and restriction sites are underlined.

Supplemental Experimental Procedures.

CRISPR array sequencing

The *Serratia* sp. ATCC39006 genome has previously been sequenced (Fineran et al., 2013), but gaps existed at type I-E and I-F CRISPR arrays (CRISPR1 and CRISPR2). The CRISPR1 and CRISPR2 arrays were amplified by PCR from the LacA strain using primer pairs PF565 + PF1834 and PF565 + PF1835, respectively. The products were gel extracted and sequenced by primer walking using primers PF565, PF633, PF1874, PF1875 or PF1905 for CRISPR1 and PF575, PF1835, PF1876, PF1903 or PF1904 for CRISPR2. Assembled reads were mapped to the existing genome (NZ_AWXH00000000) using Geneious v9 (Kearse et al., 2012). The sequenced arrays were verified by aligning short (36 nt), single-end Illumina reads from the original de novo genome assembly dataset (Fineran et al., 2013). The CRISPR3 and CRISPR4 arrays were fully assembled in the existing draft sequence.

Construction of *cas* and CRISPR reporter strains

The *cas* and CRISPR promoter regions for the type I-E, I-F and III-A systems were each cloned into an integrative *lacZ* reporter plasmid, pVIK107-Tc (Table S2). The promoter regions were amplified by PCR and cloned into pVIK107-Tc using the primers and their respective restriction endonucleases listed in Table S3. The integrative vectors were introduced into the various *Serratia* backgrounds by conjugation from the donor strain *E. coli* S17-1 λpir. Counter-selection for the *E. coli* donor was achieved using either growth on minimal medium or on LB containing appropriate antibiotics for the *Serratia* backgrounds. All integrant reporter strains were confirmed by appropriate antibiotic resistance, PCR and sequencing.

Construction of SmaR expression plasmid and heterologous repression assays

A plasmid for heterologous expression of SmaR was generated by PCR amplification of the *smaR* gene using primers PF1958 and PF1959. The product was digested with BamHI and PstI and cloned into pQE-80LoriT that was cut with the same enzymes, resulting in plasmid pPF972. *E. coli* CC118 λ*pir* was transformed with either the vector control (pQE-80LoriT) or the construct for SmaR expression (pPF972) and with either the control reporter plasmid (pVIK107-Tc) or reporter plasmids for the different *cas* and CRISPR promoter-*lacZ* fusions (Table S2). Transformants were confirmed by PCR and their antibiotic-resistance profile, and grown at 37°C for β-galactosidase assays as described above.

Construction of interference and priming plasmids

Plasmids that would be targeted or primed by the type I-E and I-F systems were generated as follows. The gene encoding GFPmut3.1 was amplified from pGREENTIR and cloned into the EcoRI and HindIII sites of pQE-80LoriT, to generate pQE-80LoriT-GFP. The gene encoding Tc^R and either no protospacer (PF1615), a type I-E protospacer (PF1617) or a type I-F protospacer containing a single nucleotide deletion at position 23 (PF1616 and PF2034) was amplified from pTRB31 (Richter et al., 2014) with the reverse primer PF210. Primers PF1617, PF1616 and PF2034 had a variable nucleotide in the type I-E and I-F PAM, respectively, which allowed for cloning of targeted and primed plasmids (see Table S3). The PCR products were digested with NcoI and XhoI and ligated into pQE-80L-oriT-GFP cut with BspHI (compatible with NcoI) and XhoI. Plasmids were sequenced and the resulting plasmids were pPF719 (no protospacer control), pPF724 (type I-E targeted), pPF722 (type I-F targeted), pPF1048 (type I-E primed), pPF1032 (type I-F primed), (Table S3). To construct the type III-A targeted plasmid, we cloned a protospacer complementary to the CRISPR3 spacer 1 into the multiple cloning region a pBAD30 expression vector derivative (pPF781). The pPF781 (naïve control) plasmid was constructed by joining a Cm^R cassette with the RP4 OriT from pQE-80LoriT (using overlap PCR with primers PF1698 + PF1700 and PF1699 + PF1701 for the first round, followed by PF1698 + PF1701 in the second round), digesting the product with KpnI and XmaI, and subsequently ligating this with a KpnI/XmaI-digested AraC/P_{BAD} fragment of pBAD30 (generated by PCR using the primers PF1666 + PF610). The protospacer fragment corresponding to CRISPR3 spacer 1 was formed by annealing primers PF2086 and PF2087, which resulted in overlapping sticky ends compatible with the KpnI and SphI sites in the multiple cloning region of pPF781. The resulting construct (pPF1043) encodes the III-A targeted protospacer on the sense strand of the transcript produced from the *araBAD* promoter.

Supplemental References

Fineran, P.C., Slater, H., Everson, L., Hughes, K., and Salmond, G.P.C. (2005). Biosynthesis of tripyrrole and beta-lactam secondary metabolites in *Serratia*: integration of quorum sensing with multiple new regulatory components in the control of prodigiosin and carbapenem antibiotic production. Mol. Microbiol. *56*, 1495–1517.

Fineran, P.C., Iglesias Cans, M.C., Ramsay, J.P., Wilf, N.M., Cossyleon, D., McNeil, M.B., Williamson, N.R., Monson, R.E., Becher, S.A., Stanton, J.-A.L., et al. (2013). Draft Genome Sequence of *Serratia* sp. strain ATCC 39006, a Model Bacterium for Analysis of the Biosynthesis and Regulation of Prodigiosin, a Carbapenem, and Gas Vesicles. Genome Announc. *1*, e01039-13.

Guzman, L.L.M., Belin, D., Carson, M.J., Beckwith, J., Luz-Maria Guzman Michael J. Carson, and Jon Beckwith, D.B. (1995). Tight Regulation, Modulation, and High-Level Expression by Vectors Containing the Arabinose PBAD Promoter. J. Bacteriol. *177*, 4121–4130.

Herrero, M., De Lorenzo, V., and Timmis, K.N. (1990). Transposon vectors containing non-antibiotic resistance selection markers for cloning and stable chromosomal insertion of foreign genes in gram-negative bacteria. J. Bacteriol. *172*, 6557–6567.

Kearse, M., Moir, R., Wilson, A., Stones-Havas, S., Cheung, M., Sturrock, S., Buxton, S., Cooper, A., Markowitz, S., Duran, C., et al. (2012). Geneious Basic: An integrated and extendable desktop software platform for the organization and analysis of sequence data. Bioinformatics *28*, 1647–1649.

de Lorenzo, V., Herrero, M., Jakubzik, U., and Timmis, K.N. (1990). Mini-Tn5 transposon derivatives for insertion mutagenesis, promoter probing, and chromosomal insertion of cloned DNA in gram-negative eubacteria. J. Bacteriol. *172*, 6568–6572.

Patterson, A.G., Chang, J.T., Taylor, C., and Fineran, P.C. (2015). Regulation of the Type I-F CRISPR-Cas system by CRP-cAMP and GalM controls spacer acquisition and interference. Nucleic Acids Res. *43*, 6038–6048.

Richter, C., Dy, R.L., McKenzie, R.E., Watson, B.N.J., Taylor, C., Chang, J.T., McNeil, M.B., Staals, R.H.J., and Fineran, P.C. (2014). Priming in the Type I-F CRISPR-Cas system triggers strand-independent spacer acquisition, bi-directionally from the primed protospacer. Nucleic Acids Res. 8516–8526.

Slater, H., Crow, M., Everson, L., and Salmond, G.P.C. (2003). Phosphate availability regulates biosynthesis of two antibiotics, prodigiosin and carbapenem, in *Serratia* via both quorum-sensing-dependent and -independent pathways. Mol. Microbiol. *47*, 303–320.

Thoma, S., and Schobert, M. (2009). An improved *Escherichia coli* donor strain for diparental mating. FEMS Microbiol. Lett. *294*, 127–132.

Thomson, N.R., Crow, M. a., McGowan, S.J., Cox, A., and Salmond, G.P.C. (2000). Biosynthesis of carbapenem antibiotic and prodigiosin pigment in *Serratia* is under quorum sensing control. Mol. Microbiol. *36*, 539–556.
