# Supplementary material for: Quorum Sensing Controls Adaptive Immunity through the Regulation of Multiple CRISPR-Cas Systems
Source: Mol Cell. 2016 Dec 15;64(6):1102–8. doi: 10.1016/j.molcel.2016.11.012 (PMC5179492; doi:10.1016/j.molcel.2016.11.012)
Supplement: Document S2. Article plus Supplemental Information [file mmc2.pdf]

Short Article

Molecular Cell

Quorum Sensing Controls Adaptive Immunity through the Regulation of Multiple CRISPR-Cas Systems

Graphical Abstract

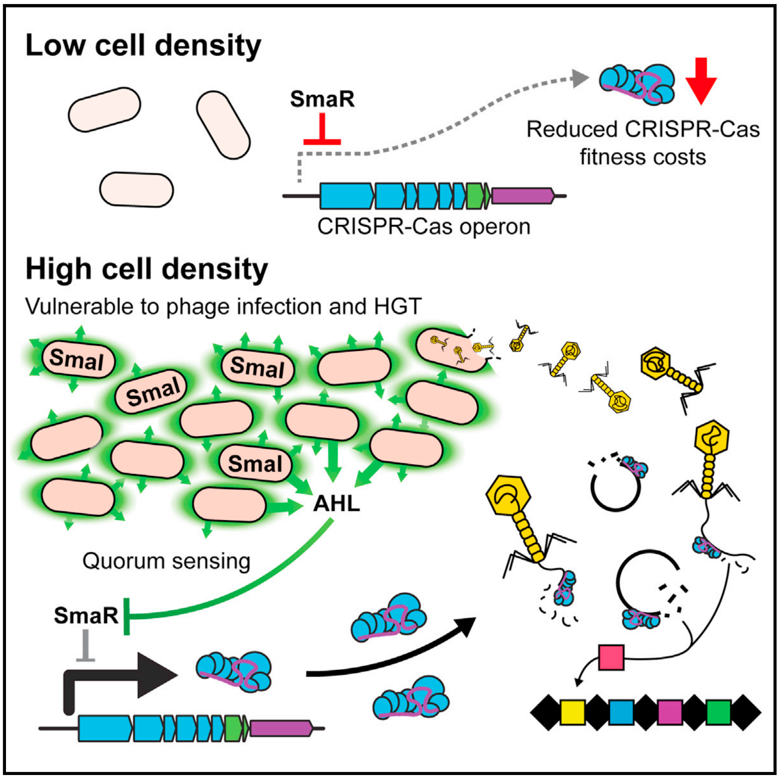

Highlights

- Quorum sensing regulates the type I-E, I-F, and III-A CRISPR-Cas systems in *Serratia*
- SmaR represses *cas* gene and CRISPR expression in the absence of AHL signals
- Both interference and adaptation are modulated by quorum sensing
- Bacteria coordinate their defenses based on cell density and the risk of infection

Authors

Adrian G. Patterson, Simon A. Jackson, Corinda Taylor, ..., Rita Przybilski, Raymond H.J. Staals, Peter C. Fineran

Correspondence

peter.fineran@otago.ac.nz

In Brief

Patterson et al. examined quorum sensing and the function of three CRISPR-Cas systems in *Serratia*. They discovered that bacteria can use chemical communication to coordinate CRISPR-Cas immune defenses at high cell densities.

Patterson et al., 2016, Molecular Cell *64*, 1102–1108
December 15, 2016 © 2016 The Authors. Published by Elsevier Inc.
http://dx.doi.org/10.1016/j.molcel.2016.11.012

Molecular Cell
Short Article

Quorum Sensing Controls Adaptive Immunity through the Regulation of Multiple CRISPR-Cas Systems

Adrian G. Patterson,[1] Simon A. Jackson,[1] Corinda Taylor,[1] Gary B. Evans,[2] George P.C. Salmond,[3] Rita Przybilski,[1] Raymond H.J. Staals,[1] and Peter C. Fineran[1,4,*]
[1]Department of Microbiology and Immunology, University of Otago, P.O. Box 56, Dunedin 9054, New Zealand
[2]Ferrier Research Institute, Victoria University of Wellington, 69 Gracefield Road, Lower Hutt 5010, New Zealand
[3]Department of Biochemistry, University of Cambridge, Tennis Court Road, Cambridge CB2 1QW, UK
[4]Lead Contact
*Correspondence: peter.fineran@otago.ac.nz
http://dx.doi.org/10.1016/j.molcel.2016.11.012

SUMMARY

Bacteria commonly exist in high cell density populations, making them prone to viral predation and horizontal gene transfer (HGT) through transformation and conjugation. To combat these invaders, bacteria possess an arsenal of defenses, such as CRISPR-Cas adaptive immunity. Many bacterial populations coordinate their behavior as cell density increases, using quorum sensing (QS) signaling. In this study, we demonstrate that QS regulation results in increased expression of the type I-E, I-F, and III-A CRISPR-Cas systems in *Serratia* cells in high-density populations. Strains unable to communicate via QS were less effective at defending against invaders targeted by any of the three CRISPR-Cas systems. Additionally, the acquisition of immunity by the type I-E and I-F systems was impaired in the absence of QS signaling. We propose that bacteria can use chemical communication to modulate the balance between community-level defense requirements in high cell density populations and host fitness costs of basal CRISPR-Cas activity.

INTRODUCTION

In nature, bacteria persist in myriad environments, from sparse populations to localized communities of high cell density, including cell chains, microcolonies, and biofilms (Hall-Stoodley et al., 2004). These bacterial populations can provide collective advantages, but a trade-off may be an increased susceptibility to bacteriophage (phage) infection (Abedon, 2012) and invasion by mobile genetic elements (Babic et al., 2011; Fuqua and Winans, 1994; Pinedo and Smets, 2005). Thus, it has been theorized that formation of microbial groups is only advantageous in times of low phage abundance, or if the threat is attenuated through elevated bacterial defenses (Abedon, 2012). It is well established that groups of bacteria regulate their behavior in response to cell density through QS, which is a widespread form of population-level communication (Miller and Bassler, 2001). As cell density increases, QS mediates accumulation of extracellular chemical signals, which are sensed by nearby bacteria, resulting in altered gene expression (Miller and Bassler, 2001).

In response to viral invasion and potentially deleterious impacts of HGT, bacteria possess an arsenal of defense systems (Dy et al., 2014; Wright et al., 2016). The CRISPR-Cas (clustered regularly interspaced short palindromic repeats [CRISPR] and their CRISPR-associated [Cas] proteins) systems provide adaptive sequence-specific immunity against foreign elements, such as phages and plasmids (Barrangou et al., 2007; Marraffini and Sontheimer, 2008). Immunity is first generated during adaptation when short invader-derived sequences (spacers) are integrated into CRISPR arrays (Amitai and Sorek, 2016; Wright et al., 2016). Second, the CRISPR arrays are transcribed and processed by Cas proteins, and in some cases host proteins, into short non-coding CRISPR RNAs (crRNAs). Finally, the crRNAs are assembled with Cas proteins into complexes that identify complementary invading nucleic acids and mediate their destruction—a process termed interference. The evolutionary success of CRISPR-Cas systems is evident from their broad distribution within bacteria and archaea (Makarova et al., 2015). However, although CRISPR-Cas systems confer tangible benefits, there are associated fitness costs (Vale et al., 2015; Westra et al., 2015). Hence, multiple systems are transcriptionally regulated (Arslan et al., 2013), which might enable physiological responsiveness to a changing environment and, thereby, a net cost-benefit balance. Since bacterial defensive requirements are predicted to change relative to population density (Abedon, 2012), we hypothesized that CRISPR-Cas immunity could be integrated into the host QS circuit, allowing increased defense at higher cell densities.

RESULTS

Expression of Three CRISPR-Cas Systems Is QS Dependent

To test the role of QS in CRISPR-Cas regulation, we used *Serratia* sp. ATCC39006, which possesses a LuxIR-type QS system (Thomson et al., 2000) and three CRISPR-Cas systems (type I-E, I-F, and III-A), each with at least one CRISPR array (Figure 1A).

 © 2016 The Authors. Published by Elsevier Inc.
This is an open access article under the CC BY license (http://creativecommons.org/licenses/by/4.0/).

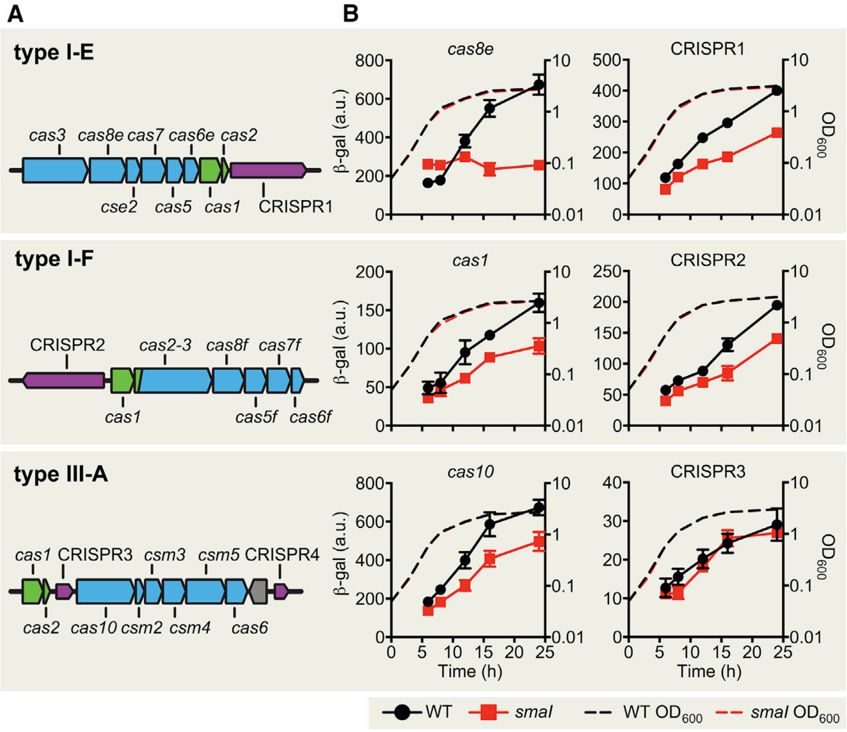

Figure 1. Quorum Sensing Regulates Expression of Three Distinct CRISPR-Cas Systems

(A) Schematic of the *Serratia* sp. ATCC39006 CRISPR-Cas systems. Genes encoding interference or adaptation machinery are colored blue or green, respectively. The four CRISPR arrays—CRISPR1 (I-E, 52 spacers), CRISPR2 (I-F, 57 spacers), CRISPR3 (III-A, 9 spacers), and CRISPR4 (III-A, 8 spacers)—are colored purple.

(B) *cas*::*lacZ* and CRISPR::*lacZ* activity and growth for each of the type I-E, type I-F, and type III-A reporter strains in the WT and *smaI* mutant backgrounds (Table S1). Differences in activity between WT and *smaI* beyond 12 hr were statistically significant ($p \leq 0.05$) for each reporter except CRISPR3 (two-way analysis of variance [ANOVA] with Bonferroni's multiple comparisons test). Data shown are the mean ± SD (n = 3). Figure S1 contains data for *smaI*::*lacZ* expression and C4-HSL production in addition to type I-E *cas3* and type III-A *cas1* and CRISPR4::*lacZ* expression. Complementation of all CRISPR-Cas reporters with C4-HSL is shown in Figure S1.

Quorum sensing in Gram-negative bacteria typically utilizes LuxI family proteins to generate *N*-acyl homoserine lactone (AHL) signals, which are sensed by LuxR-type transcriptional regulators (Miller and Bassler, 2001). In *Serratia*, the *luxIR* homologs, *smaI* and *smaR*, control secondary metabolite production and motility, and SmaI produces predominantly *N*-butanoyl-L-homoserine lactone (C4-HSL) (Fineran et al., 2005; Thomson et al., 2000). Under our experimental conditions, the transcription of both *smaI* and AHL levels rose as cell densities increased, peaking at late exponential growth as cultures transitioned into stationary phase (Figure S1). To examine the effects of QS on CRISPR-Cas, we assessed *cas* operon and CRISPR expression in the wild-type (WT) and a signal-deficient *smaI* mutant throughout growth (Figures 1B and S1). Remarkably, expression of *cas* operons for all three CRISPR-Cas systems, as well as CRISPR1 (type I-E) and CRISPR2 (type I-F), was significantly reduced in the absence of AHL signal production (Figure 1B). The CRISPR arrays associated with the type III-A system (CRISPR3 and CRISPR4) exhibited low expression in the WT and were not regulated by QS since no further reduction was detected in the *smaI* mutant (Figures 1B and S1). We were able to fully complement the *smaI* mutant throughout growth by the addition of chemically synthesized C4-HSL, thereby confirming that the decreased *cas* and CRISPR expression in the *smaI* mutant resulted from the lack of AHL production (Figure S1). In agreement with previous work examining QS controlled secondary metabolite production in *Serratia*, addition of C4-HSL did not induce precocious induction of gene expression in the WT (Slater et al., 2003). Overall, expression of one or both core components (*cas* genes or CRISPRs) from all three CRISPR-Cas systems was subject to QS control.

CRISPR-Cas Regulation Involves the SmaR Repressor

In the absence of the AHLs, the SmaR transcriptional regulator acts as a DNA-binding repressor (Fineran et al., 2005; Slater et al., 2003; Thomson et al., 2000). At increased cell density, AHLs accumulate and bind SmaR, thereby inhibiting its DNA binding activity, resulting in elevated gene expression through a de-repression mechanism (Fineran et al., 2005). Mutation of *smaR* alone had no effect on *cas* and CRISPR expression throughout growth (Figures 2 and S2). The lack of enhanced expression in the *smaR* mutant is well established for genes previously shown to be controlled by QS in *Serratia* and is likely to be due to other required physiological and regulatory inputs (Fineran et al., 2005). Deletion of *smaR* in the *smaI* mutant restored expression of the *cas* operons and CRISPR arrays throughout growth (Figures 2 and S2), demonstrating that, in the absence of AHL production, SmaR acts as a repressor of CRISPR and *cas* gene expression. In agreement, plasmid-encoded SmaR caused significantly reduced expression from each of the QS-regulated CRISPR and *cas* promoters but not from a non-QS regulated control promoter (Figure S3). The SmaR-mediated repression observed using this system was similar to the reduction in CRISPR and *cas* expression upon deletion of *smaI* in *Serratia*. Therefore, these results demonstrate that SmaR represses CRISPR-Cas expression in the absence of the QS signaling molecules.

Quorum Sensing Modulates CRISPR Interference

Evidence that the CRISPR-Cas modules were regulated by the host QS circuit supported our hypothesis that defense against invaders would be elevated at high cell densities. To determine whether the transcriptional changes correlated with modulation of immunity, we exposed *Serratia* cells growing in high-density populations to donor bacteria that transfer, via conjugation, plasmids that mimicked invaders that were encountered previously.

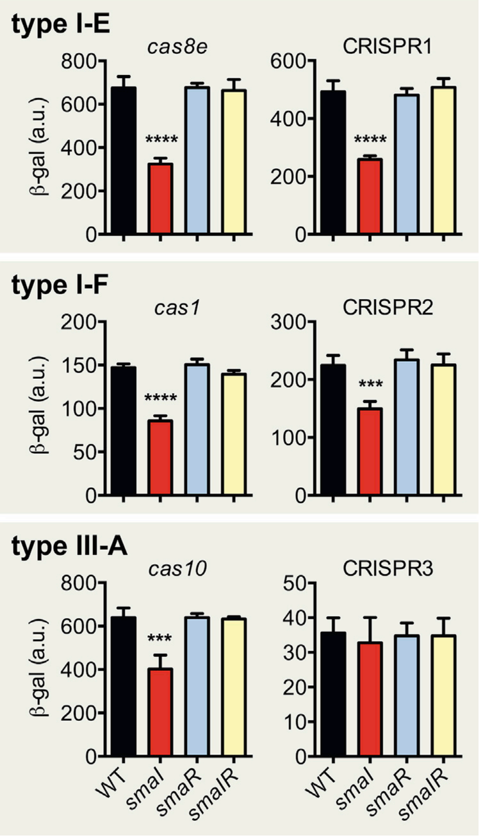

Figure 2. SmaR Represses CRISPR-Cas Expression in the Absence of QS Signals

cas::*lacZ* and CRISPR::*lacZ* activity for each of the type I-E, I-F, and III-A reporter strains in the WT, *smaI* mutant, *smaR* mutant, and *smaIR* mutant backgrounds (Table S1) at 24 hr post inoculation. Statistical significance was calculated by one-way ANOVA with the Bonferroni's multiple comparisons test (***p ≤ 0.001, ****p ≤ 0.0001). Data shown are the mean ± SD (n = 3). Expression of all reporters, including CRISPR4 activity, is shown throughout growth in Figure S2. Repression of the CRISPR-Cas reporters by expression of SmaR is shown in Figure S3.

These plasmids contained sequences complementary to the first spacer present in CRISPR1, CRISPR2, or CRISPR3 for the type I-E, I-F, and III-A systems, respectively (Table S2). These target sequences are termed protospacers and, for the type I-E and I-F systems, included canonical protospacer adjacent motif (PAM) sequences that are necessary to evoke direct interference. In the WT populations, all three CRISPR-Cas systems were capable of robust interference of the respective target plasmids but not of untargeted control plasmids (Figure 3), demonstrating that each native system is functional. The conjugation efficiencies of untargeted (naive) control plasmids for the *smaI* mutant were comparable to the WT, demonstrating that there were no CRISPR-Cas-independent effects in this background. In contrast, the interference capability was significantly reduced in signaling-deficient populations (the *smaI* mutant) by ~20-fold for type I-E, ~500-fold for type I-F, and ~240-fold for type III-A targeting (Figure 3). Unexpectedly, the type I-E system showed the weakest interference response to QS, despite having the strongest effect on the *cas8e* promoter (Figure 1). It is likely that the activity of other type I-E components might form a bottleneck for the overall level of interference, which is the case for *cas3* in the *E. coli* type I-E system (Majsec et al., 2016). The impaired interference in all three CRISPR-Cas systems could be rescued via the addition of exogenous QS signal (Figure S4). Despite the reduced levels of interference in the *smaI* mutant, we still observed relatively efficient recognition and destruction of the targeted plasmids by each of the CRISPR-Cas systems. Together, these results demonstrate that QS signaling modulates the efficiency of interference and is necessary to allow enhanced defense at high cell densities.

Quorum Sensing Regulates Spacer Acquisition

Adaptation is a critical function of CRISPR-Cas systems, allowing generation of new immunity through spacer acquisition (Amitai and Sorek, 2016; Wright et al., 2016). Therefore, we asked whether this aspect of CRISPR-Cas was also regulated by QS. Two adaptation modes are known, naive and primed (Amitai and Sorek, 2016; Wright et al., 2016). During naive adaptation, spacers are acquired from elements to which no previous immunity exists, whereas primed adaptation, observed in type I systems, enhances acquisition of spacers from elements resembling those previously encountered (Datsenko et al., 2012). To examine adaptation in the WT and the *smaI* mutant, we tested their abilities to acquire spacers from either a "naive" plasmid, representing an unrecognized invader, or "primed" plasmids, representing escape mutants from targets of the type I-E or type I-F systems. Primed plasmids contained non-consensus PAMs to trigger primed acquisition of additional spacers (Table S2). For WT cells containing the naive plasmid, repeated passage to high cell density in the absence of antibiotic selection yielded no detectable naive spacer acquisition (Figure 4). In contrast, primed spacer acquisition from the escape plasmids was readily observed in the WT for both the type I-E and type I-F systems. Adaptation in the *smaI* mutant was reduced by ~75% and ~80% for the type I-E and type I-F systems, respectively (Figure 4). The impaired adaptation in both CRISPR-Cas systems was rescued via the addition of exogenous QS signal (Figure S4). In summary, QS-mediated elevation of CRISPR-Cas activity enhances the generation of immunological memory within high-density populations by promoting increased spacer acquisition.

DISCUSSION

Here we demonstrate that in a single strain, the expression of three different CRISPR-Cas systems (including types I and III) is regulated by the host QS circuit to significantly modulate immunity, including both interference and adaptation. The QS effect on adaptation is highly relevant with respect to bacterial population fitness because increased diversity of CRISPR spacers within communities restricts the success of phage escape mutants (van Houte et al., 2016). We show that QS-defective populations generate fewer new spacers during adaptation, hence less diversity, thereby highlighting the importance of cell-cell communication in stimulating population-level CRISPR-Cas resistance. This nascent immunity is further

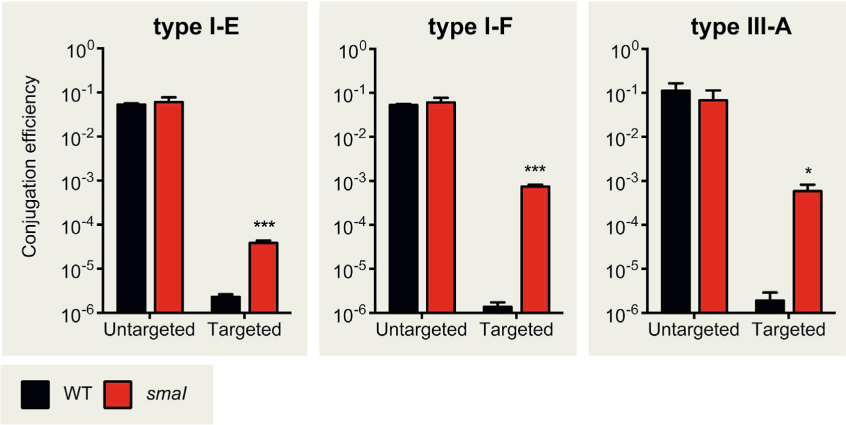

Figure 3. Quorum Sensing Is Required for Heightened CRISPR-Cas Interference

Conjugation efficiency of untargeted plasmids or plasmids targeted by the type I-E, type I-F, or type III-A systems in the WT or *smaI* mutant backgrounds. Conjugation efficiency was scored as transconjugants/recipients. Statistical significance was assessed by unpaired two-tailed t test (*$p \leq 0.05$, ***$p \leq 0.001$). Data shown are the mean ± SD (n = 3). Complementation of all phenotypes using C4-HSL is shown in Figure S4.

reinforced by the elevated interference invoked by spacers from the three different systems.

Our results demonstrate the importance of cell signaling in coordinating adaptive immunity when microbial groups are at high cell densities. Since HGT frequency, or phage spread, is less likely at low population densities (Abedon, 2012; Pinedo and Smets, 2005), defense does not need to be high, but it is still necessary. In agreement, we still observed efficient, albeit significantly reduced, CRISPR-Cas immunity under conditions mimicking low cell densities (i.e., the *smaI* mutant). Since SmaR is a repressor that is inactivated by AHLs, this particular QS system can be viewed as dampening down immunity at low cell density. The burden of CRISPR-Cas systems, such as lethal auto-immunity caused by self-targeting (Staals et al., 2016; Vercoe et al., 2013; Stern et al., 2010), might have provided selective pressure to evolve this "suppress when least required" mechanism. In contrast, successful phage infection of high cell density bacterial communities results in high localized viral loads that might overwhelm basal level CRISPR-Cas defenses. Thus, upregulation of CRISPR-Cas via QS facilitates transition to a heightened defensive state that is better suited to cope with high multiplicity of infection phage attacks.

As well as increasing general defense against invading elements, upregulation of CRISPR-Cas activity might also allow for an enhanced response to the stimulation of HGT or prophage release that can be triggered by QS. For example, diverse mobile elements coordinate their dissemination via QS, including AHL-based control of conjugative Ti plasmid transfer in *Agrobacterium tumefaciens* (Fuqua and Winans, 1994). Furthermore, many QS signals, including AHLs, *Pseudomonas* quinolone signal (PQS), and AI-2, can induce prophage induction in Gram-positive and Gram-negative bacteria (Fernández-Piñar et al., 2011; Hargreaves et al., 2014; Rossmann et al., 2015). It is salient that we observed QS-dependent regulation of type III-A activity. Type III are the only known CRISPR-Cas systems to target DNA in a transcription-dependent manner, which is thought to protect bacteria from active or induced prophages, while minimizing self-targeting of integrated prophages (Goldberg et al., 2014). Therefore, the QS-dependent response of the type III-A system might not only protect from phage infection, but also restrict the proliferation of viral progeny during prophage induction. Interestingly, a recent global metagenomic study highlighted the relevance of temperate phages in ecosystems with high bacterial abundances (Knowles et al., 2016), implying that the role of QS in prophage induction and CRISPR-Cas regulation could be ecologically significant.

The broad distribution of both CRISPR-Cas and QS systems within diverse bacteria suggests that QS-dependent regulation of immunity should be widespread. In support of this, we performed an analysis of published microarray data from *Pectobacterium atrosepticum* and discovered a significant reduction in type I-F *cas* gene expression in an AHL synthase mutant (*expI*) that was most pronounced at high cell density (Bowden et al., 2013). Likewise, in a *Burkholderia glumae* transcriptomic study, mutation of *luxI* homologs resulted in decreased expression of type I-F *cas* genes (Gao et al., 2015). Quorum sensing mechanisms used by bacteria are diverse, with peptide pheromones common in Gram-positives and the "universal" AI-2 signal produced by LuxS in many disparate bacteria. Furthermore, multiple CRISPR-Cas types (e.g., I-E, I-F, and III-A in *Serratia*) can be connected to QS circuits. Therefore, we predict that the control of adaptive immunity by QS is likely to be widespread across diverse bacteria and CRISPR-Cas types, irrespective of the precise signaling mechanism.

Fittingly, other phage defense systems operate under QS control (Høyland-Kroghsbo et al., 2013; Kolodkin-Gal et al., 2007). For example, an AHL-dependent reduction in receptors on *E. coli* limits infection by λ and χ phages (Høyland-Kroghsbo et al., 2013). Since *E. coli* does not produce AHLs, but has a LuxR sensor (SdiA), this might provide protection against broad host-range phages preying on neighboring bacteria. Within heterogeneous populations, analogous cross-species QS signaling could boost the CRISPR-Cas defenses of minority species, reducing the risk these individuals pose as vectors or reservoirs for phage spread. Other defense strategies provide population-level protection, such as abortive infection systems, which typically result in the "altruistic" suicide of infected cells. One such system, *mazEF* from *E. coli*, is regulated by a QS pentapeptide and limits phage P1 (Kolodkin-Gal et al., 2007). These suicidal defenses are most successful when bacteria are growing with spatial structure (Fukuyo et al., 2012). It is interesting that both abortive infection and CRISPR-Cas are most effective in populations at high cell density.

Because QS is an important regulator of CRISPR-Cas, invaders are likely to have evolved evasion mechanisms. In

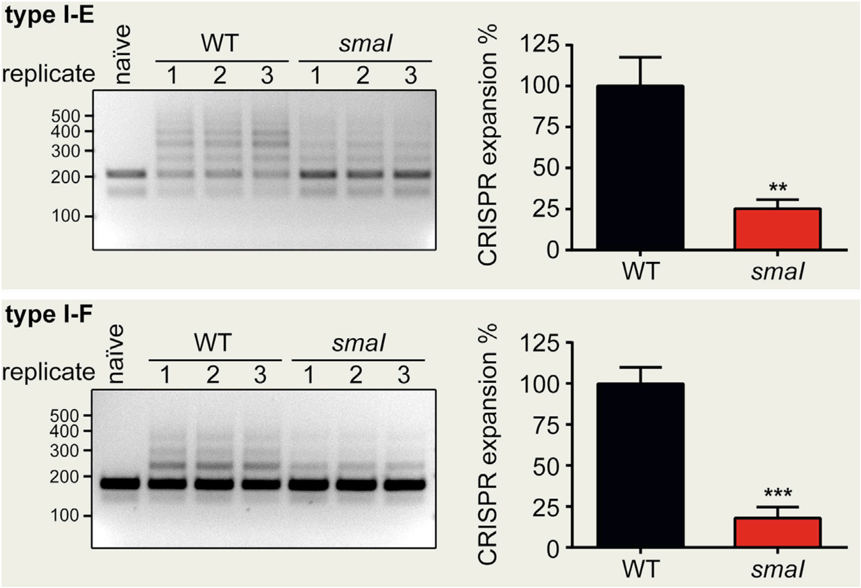

Figure 4. Quorum Sensing Boosts Adaptation in the Type I CRISPR-Cas Systems
Spacer acquisition (CRISPR expansion) for the WT and *smaI* mutant strains was quantified after exposure to primed plasmids for either the type I-E or type I-F systems. The WT strain with the naive plasmid is also shown. CRISPR arrays were amplified by PCR and analyzed on 3% agarose gels. CRISPR expansion was normalized relative to the expansion observed in the WT (WT mean set as 100%). Statistical significance was assessed by unpaired two-tailed t test (**p ≤ 0.01, ***p ≤ 0.001). Data shown are the mean ± SD (n = 3). Complementation of all phenotypes using C4-HSL is shown in Figure S4.

agreement, some phages encode acylases, enzymes that degrade AHLs, and others encode their own QS systems (Hargreaves et al., 2014). These phages might block or interfere with QS to improve their reproductive success in the face of CRISPR-Cas competition—akin to anti-CRISPR proteins (Bondy-Denomy et al., 2013). Indeed, phages have been engineered to express AHL-degrading enzymes that enhance their ability to disrupt biofilms by eliciting cell death and by inhibiting QS (Pei and Lamas-Samanamud, 2014). Our findings suggest that phage therapies that are combined with anti-QS strategies (e.g., engineered phages or anti-QS molecules) might assist in the evasion of CRISPR-Cas defense during treatment.

EXPERIMENTAL PROCEDURES

Culture Conditions, Strains, and Plasmids

Tables S1 and S2 list all strains and plasmids used in this study, respectively, and Table S3 lists the oligonucleotides used. Details of strain and plasmid constructions are provided in the Supplemental Experimental Procedures. Unless otherwise stated, *Serratia* sp. ATCC39006 strains were grown at 30°C and *E. coli* strains at 37°C in Lysogeny Broth (LB), minimal medium agar (0.1% w/v $(NH_4)_2SO_4$, 0.41 mM $MgSO_4$, 0.2% w/v glucose, 40 mM K_2HPO_4, 14.7 mM KH_2PO_4 [pH 6.9–7.1], 1.5% w/v agar) or on LB-agar (LBA) plates containing 1.5% (w/v) agar. When required, media were supplemented with antibiotics as follows: ampicillin (Ap; 100 μg/mL), chloramphenicol (Cm; 25 μg/mL), kanamycin (Km; 50 μg/mL), spectinomycin (Sp; 50 μg/mL), and tetracycline (Tc; 10 μg/mL). 5-aminolevulinic acid (Ala; 50 μg/mL) was added for growth of ST18. Bacterial growth was measured in a Jenway 6300 Spectrophotometer at 600 nm (OD_{600}), except when grown in 96-well microtiter plates, where it was measured in a Varioskan Flash Multimode Reader (Thermo Fisher Scientific) at 600 nm. All experiments were repeated in at least three biological replicates.

AHL Production Assay

AHL production was assessed using bioassay plates as previously described (McClean et al., 1997). Briefly, the bioassay plates were prepared by seeding 100 mL of molten 0.75% LBA overlay with 1 mL of an ISTSO4 overnight culture, which was then poured over the surface of 1.5% LBA in a 25 cm × 25 cm square petri dish. Once set, holes were punched in the agar using a sterile cork borer. Samples for assay of AHL production were taken from cultures of WT (LacA) *Serratia* grown in multiple 1 mL aliquots of LB media in a 96 square deep-well plate (Labcon) incubated at 1,200 rpm at 30°C in a microplate shaker (BioProducts Incumix). Samples (1 mL) were pelleted by centrifugation at 13,000 rpm for 4 min, and the supernatants were sterilized using 0.22 μM syringe filters (Millipore). Supernatant samples for each time point were then used to fill the wells in the bioassay plate. The plate was subsequently incubated at 30°C for 24 hr, and the area of pigmentation surrounding each well was measured and reported as arbitrary units (a.u.). Supernatants from *smaI* mutant cultures were included at each time point and did not produce any detectable AHL.

β-Galactosidase Expression Assays

Growth of bacterial strains containing the *lacZ* reporters and the β-galactosidase assays were performed as previously described (Patterson et al., 2015). The reporter strains contained a single chromosomal integration of the *lacZ* reporter fused to the ATG start codon of the various *cas* genes in the native genetic context, or to the start of the different CRISPR arrays. Therefore, they report on the expression from the various genes or arrays from their natural promoter positions within the chromosome (for a schematic see Figure S1 in Patterson et al., 2015). Briefly, bacteria were grown in 1 mL of LB with Tc in 96 square deep-well plates (Labcon) and incubated in a microplate shaker (BioProducts Incumix) at 1,200 rpm and 30°C. β-galactosidase assays were performed in a Varioskan Flash Multimode Reader (Thermo Fisher Scientific) as described previously using the fluorogenic substrate (4-Methylumbelliferyl β-D-galactoside; MUG) (Patterson et al., 2015; Ramsay, 2013). Relative fluorescent units (RFUs) per second were calculated using the linear increase in fluorescence, which was normalized to the OD_{600} of the sample (RFU/s/OD_{600}). RFU/s/OD_{600} measurements are depicted as arbitrary units in the axis labels of relevant figures.

Chemical Complementation Using *N*-Butanoyl-L-Homoserine Lactone

N-butanoyl-L-homoserine lactone (C4-HSL) was synthesized as described previously (Hodgkinson et al., 2011) and the chemical nature confirmed (Geske et al., 2005). C4-HSL was stored in dimethyl sulfoxide (DMSO) and was added at a final concentration of 0.5 μM at the start of growth in the chemical complementation experiments. In the control samples, an equivalent volume of DMSO was added as a solvent control. Growth and β-galactosidase assays were performed as described earlier. For complementation of interference and adaptation experiments, assays were performed as described later, but all plates and bacterial cultures were supplemented with 0.5 μM C4-HSL or DMSO (control).

Conjugation Efficiency Assays

Conjugation efficiency was assessed in a similar manner to that described previously (Patterson et al., 2015; Richter et al., 2014). *E. coli* ST18 were used as donors for the conjugation of control (pPF719) and type I-E (pPF724) or type I-F

(pPF722) targeted plasmids, or of control (pPF781) and type III-A (pPF1043) targeted plasmids. Plasmids pPF724, pPF722, and pPF1043 each contain a protospacer targeted by spacer 1 from either CRISPR1 (type I-E), CRISPR2 (type I-F), or CRISPR3 (type III-A), respectively. Recipient strains were WT (LacA) and *smaI* (LIS). Strains were grown overnight in LB with appropriate antibiotics, the OD_{600} adjusted to 1 and washed twice with LB. Donors and recipients were mixed in a 1:1 ratio, and 5 μL was spotted on 0.2 μm filters (Millipore) on LBA + Ala and incubated for 24 hr. Next, the filters were added to 2 mL PBS, the bacteria were resuspended, and dilution series were plated onto LB for recipient counts or with the addition of antibiotics for selection of transconjugant counts. For the type III-A experiments either 20 mM glucose or 0.02% arabinose was included in the plates for the filter matings or transconjugant selection, respectively. In all cases, conjugation efficiency was calculated as transconjugants per recipients.

Adaptation Assays

Plasmids pPF719 (non-targeted "naïve" control), pPF1048 ("primed" type I-E), and pPF1032 ("primed" type I-F) were transferred from *E. coli* ST18 by conjugation into WT (LacA) and *smaI* (LIS) strains and plated on LBA + Tc. After PCR confirmation of the transconjugants, overnight cultures of each strain grown in the presence of Tc were used to inoculate fresh 5 mL cultures in LB without antibiotics in 20 mL universals. These were then incubated at 30°C with shaking and passaged for 6 days by daily transfer of 10 μL to 5 mL of fresh LB. CRISPR expansion (indicative of spacer acquisition) was determined by PCR directly on cells from passaged cultures (DreamTaq, Thermo Fisher Scientific) using primers PF1887 + PF1989 for CRISPR1 and PF1888 + PF1990 for CRISPR2. PCR products were separated by 3% agarose gel electrophoresis and stained with ethidium bromide, and spacer acquisition was quantified using ImageJ (Schneider et al., 2012).

ACCESSION NUMBERS

Unprocessed image files used to prepare the figures in this manuscript have been deposited in Mendeley Data and are available at http://dx.doi.org/10.17632/x55v9zf59x.1.

SUPPLEMENTAL INFORMATION

Supplemental Information includes Supplemental Experimental Procedures, four figures, and three tables and can be found with this article online at http://dx.doi.org/10.1016/j.molcel.2016.11.012.

AUTHOR CONTRIBUTIONS

Conceptualization, A.G.P., S.A.J., R.P., R.H.J.S., and P.C.F. Investigation, A.G.P., S.A.J., C.T., R.P., and R.H.J.S. Resources, G.B.E. and G.P.C.S. Formal Analysis, A.G.P., S.A.J., G.P.C.S., R.P., R.H.J.S., and P.C.F. Writing – Original Draft, A.G.P., S.A.J., R.H.J.S., and P.C.F. Writing – Review & Editing, A.G.P., S.A.J., G.B.E., G.P.C.S., R.P., R.H.J.S., and P.C.F. Funding Acquisition and Supervision, P.C.F.

ACKNOWLEDGMENTS

This work was supported by a Rutherford Discovery Fellowship (P.C.F.) from the Royal Society of New Zealand (RSNZ) and the Marsden Fund, RSNZ. A.G.P. was supported by a University of Otago Doctoral Scholarship. G.P.C.S. is funded by the Biotechnology and Biological Sciences Research Council, UK. We thank members of the P.C.F. laboratory for helpful discussions, Steven Bowden for providing analyzed *P. atrosepticum* microarray data, and Andrew Gray for statistical advice.

Received: September 16, 2016
Revised: October 7, 2016
Accepted: November 4, 2016
Published: November 17, 2016

REFERENCES

Abedon, S.T. (2012). Spatial vulnerability: bacterial arrangements, microcolonies, and biofilms as responses to low rather than high phage densities. Viruses *4*, 663–687.

Amitai, G., and Sorek, R. (2016). CRISPR-Cas adaptation: insights into the mechanism of action. Nat. Rev. Microbiol. *14*, 67–76.

Arslan, Z., Westra, E.R., Wagner, R., and Pul, Ü. (2013). Regulation of CRISPR-Based Immune Responses. In CRISPR-Cas Systems, R. Barrangou and J. van der Oost, eds. (Springer), pp. 93–113.

Babic, A., Berkmen, M.B., Lee, C.A., and Grossman, A.D. (2011). Efficient gene transfer in bacterial cell chains. MBio *2*, e00027–e11.

Barrangou, R., Fremaux, C., Deveau, H., Richards, M., Boyaval, P., Moineau, S., Romero, D.A., and Horvath, P. (2007). CRISPR provides acquired resistance against viruses in prokaryotes. Science *315*, 1709–1712.

Bondy-Denomy, J., Pawluk, A., Maxwell, K.L., and Davidson, A.R. (2013). Bacteriophage genes that inactivate the CRISPR/Cas bacterial immune system. Nature *493*, 429–432.

Bowden, S.D., Eyres, A., Chung, J.C.S., Monson, R.E., Thompson, A., Salmond, G.P.C., Spring, D.R., and Welch, M. (2013). Virulence in *Pectobacterium atrosepticum* is regulated by a coincidence circuit involving quorum sensing and the stress alarmone, (p)ppGpp. Mol. Microbiol. *90*, 457–471.

Datsenko, K.A., Pougach, K., Tikhonov, A., Wanner, B.L., Severinov, K., and Semenova, E. (2012). Molecular memory of prior infections activates the CRISPR/Cas adaptive bacterial immunity system. Nat. Commun. *3*, 945.

Dy, R.L., Richter, C., Salmond, G.P.C., and Fineran, P.C. (2014). Remarkable Mechanisms in Microbes to Resist Phage Infections. Annu Rev Virol *1*, 307–331.

Fernández-Piñar, R., Cámara, M., Dubern, J.F., Ramos, J.L., and Espinosa-Urgel, M. (2011). The *Pseudomonas aeruginosa* quinolone quorum sensing signal alters the multicellular behaviour of *Pseudomonas putida* KT2440. Res. Microbiol. *162*, 773–781.

Fineran, P.C., Slater, H., Everson, L., Hughes, K., and Salmond, G.P.C. (2005). Biosynthesis of tripyrrole and beta-lactam secondary metabolites in *Serratia*: integration of quorum sensing with multiple new regulatory components in the control of prodigiosin and carbapenem antibiotic production. Mol. Microbiol. *56*, 1495–1517.

Fukuyo, M., Sasaki, A., and Kobayashi, I. (2012). Success of a suicidal defense strategy against infection in a structured habitat. Sci. Rep. *2*, 238.

Fuqua, W.C., and Winans, S.C. (1994). A LuxR-LuxI type regulatory system activates *Agrobacterium* Ti plasmid conjugal transfer in the presence of a plant tumor metabolite. J. Bacteriol. *176*, 2796–2806.

Gao, R., Krysciak, D., Petersen, K., Utpatel, C., Knapp, A., Schmeisser, C., Daniel, R., Voget, S., Jaeger, K.-E., and Streit, W.R. (2015). Genome-wide RNA sequencing analysis of quorum sensing-controlled regulons in the plant-associated *Burkholderia glumae* PG1 strain. Appl. Environ. Microbiol. *81*, 7993–8007.

Geske, G.D., Wezeman, R.J., Siegel, A.P., and Blackwell, H.E. (2005). Small molecule inhibitors of bacterial quorum sensing and biofilm formation. J. Am. Chem. Soc. *127*, 12762–12763.

Goldberg, G.W., Jiang, W., Bikard, D., and Marraffini, L.A. (2014). Conditional tolerance of temperate phages via transcription-dependent CRISPR-Cas targeting. Nature *514*, 633–637.

Hall-Stoodley, L., Costerton, J.W., and Stoodley, P. (2004). Bacterial biofilms: from the natural environment to infectious diseases. Nat. Rev. Microbiol. *2*, 95–108.

Hargreaves, K.R., Kropinski, A.M., and Clokie, M.R. (2014). Bacteriophage behavioral ecology: How phages alter their bacterial host's habits. Bacteriophage *4*, e29866.

Hodgkinson, J.T., Galloway, W.R.J.D., Casoli, M., Keane, H., Su, X., Salmond, G.P.C., Welch, M., and Spring, D.R. (2011). Robust routes for the synthesis of

N-acylated-L-homoserine lactone (AHL) quorum sensing molecules with high levels of enantiomeric purity. Tetrahedron Lett. *52*, 3291–3294.

Høyland-Kroghsbo, N.M., Maerkedahl, R.B., and Svenningsen, S.L. (2013). A quorum-sensing-induced bacteriophage defense mechanism. MBio *4*, e00362–e12.

Knowles, B., Silveira, C.B., Bailey, B.A., Barott, K., Cantu, V.A., Cobián-Güemes, A.G., Coutinho, F.H., Dinsdale, E.A., Felts, B., Furby, K.A., et al. (2016). Lytic to temperate switching of viral communities. Nature *531*, 466–470.

Kolodkin-Gal, I., Hazan, R., Gaathon, A., Carmeli, S., and Engelberg-Kulka, H. (2007). A linear pentapeptide is a quorum-sensing factor required for *mazEF*-mediated cell death in *Escherichia coli*. Science *318*, 652–655.

Majsec, K., Bolt, E.L., and Ivančić-Baće, I. (2016). Cas3 is a limiting factor for CRISPR-Cas immunity in *Escherichia coli* cells lacking H-NS. BMC Microbiol. *16*, 28.

Makarova, K.S., Wolf, Y.I., Alkhnbashi, O.S., Costa, F., Shah, S.A., Saunders, S.J., Barrangou, R., Brouns, S.J., Charpentier, E., Haft, D.H., et al. (2015). An updated evolutionary classification of CRISPR-Cas systems. Nat. Rev. Microbiol. *13*, 722–736.

Marraffini, L.A., and Sontheimer, E.J. (2008). CRISPR interference limits horizontal gene transfer in staphylococci by targeting DNA. Science *322*, 1843–1845.

McClean, K.H., Winson, M.K., Fish, L., Taylor, A., Chhabra, S.R., Camara, M., Daykin, M., Lamb, J.H., Swift, S., Bycroft, B.W., et al. (1997). Quorum sensing and *Chromobacterium violaceum*: exploitation of violacein production and inhibition for the detection of N-acylhomoserine lactones. Microbiology *143*, 3703–3711.

Miller, M.B., and Bassler, B.L. (2001). Quorum sensing in bacteria. Annu. Rev. Microbiol. *55*, 165–199.

Patterson, A.G., Chang, J.T., Taylor, C., and Fineran, P.C. (2015). Regulation of the Type I-F CRISPR-Cas system by CRP-cAMP and GalM controls spacer acquisition and interference. Nucleic Acids Res. *43*, 6038–6048.

Pei, R., and Lamas-Samanamud, G.R. (2014). Inhibition of biofilm formation by T7 bacteriophages producing quorum-quenching enzymes. Appl. Environ. Microbiol. *80*, 5340–5348.

Pinedo, C.A., and Smets, B.F. (2005). Conjugal TOL transfer from *Pseudomonas putida* to *Pseudomonas aeruginosa*: effects of restriction proficiency, toxicant exposure, cell density ratios, and conjugation detection method on observed transfer efficiencies. Appl. Environ. Microbiol. *71*, 51–57.

Ramsay, J.P. (2013). High-throughput β-galactosidase and β-glucuronidase Assays Using Fluorogenic Substrates. Bio-protocol. *3*, e827.

Richter, C., Dy, R.L., McKenzie, R.E., Watson, B.N.J., Taylor, C., Chang, J.T., McNeil, M.B., Staals, R.H.J., and Fineran, P.C. (2014). Priming in the Type I-F CRISPR-Cas system triggers strand-independent spacer acquisition, bi-directionally from the primed protospacer. Nucleic Acids Res. *42*, 8516–8526.

Rossmann, F.S., Racek, T., Wobser, D., Puchalka, J., Rabener, E.M., Reiger, M., Hendrickx, A.P.A., Diederich, A.K., Jung, K., Klein, C., and Huebner, J. (2015). Phage-mediated dispersal of biofilm and distribution of bacterial virulence genes is induced by quorum sensing. PLoS Pathog. *11*, e1004653.

Schneider, C.A., Rasband, W.S., and Eliceiri, K.W. (2012). NIH Image to ImageJ: 25 years of image analysis. Nat. Methods *9*, 671–675.

Slater, H., Crow, M., Everson, L., and Salmond, G.P.C. (2003). Phosphate availability regulates biosynthesis of two antibiotics, prodigiosin and carbapenem, in *Serratia* via both quorum-sensing-dependent and -independent pathways. Mol. Microbiol. *47*, 303–320.

Staals, R.H.J., Jackson, S.A., Biswas, A., Brouns, S.J.J., Brown, C.M., and Fineran, P.C. (2016). Interference-driven spacer acquisition is dominant over naive and primed adaptation in a native CRISPR-Cas system. Nat. Commun. *7*, 12853.

Stern, A., Keren, L., Wurtzel, O., Amitai, G., and Sorek, R. (2010). Self-targeting by CRISPR: gene regulation or autoimmunity? Trends Genet. *26*, 335–340.

Thomson, N.R., Crow, M.A., McGowan, S.J., Cox, A., and Salmond, G.P.C. (2000). Biosynthesis of carbapenem antibiotic and prodigiosin pigment in *Serratia* is under quorum sensing control. Mol. Microbiol. *36*, 539–556.

Vale, P.F., Lafforgue, G., Gatchitch, F., Gardan, R., Moineau, S., and Gandon, S. (2015). Costs of CRISPR-Cas-mediated resistance in *Streptococcus thermophilus*. Proc. Biol. Sci. *282*, 20151270.

van Houte, S., Ekroth, A.K.E., Broniewski, J.M., Chabas, H., Ashby, B., Bondy-Denomy, J., Gandon, S., Boots, M., Paterson, S., Buckling, A., and Westra, E.R. (2016). The diversity-generating benefits of a prokaryotic adaptive immune system. Nature *532*, 385–388.

Vercoe, R.B., Chang, J.T., Dy, R.L., Taylor, C., Gristwood, T., Clulow, J.S., Richter, C., Przybilski, R., Pitman, A.R., and Fineran, P.C. (2013). Cytotoxic chromosomal targeting by CRISPR/Cas systems can reshape bacterial genomes and expel or remodel pathogenicity islands. PLoS Genet. *9*, e1003454.

Westra, E.R., van Houte, S., Oyesiku-Blakemore, S., Makin, B., Broniewski, J.M., Best, A., Bondy-Denomy, J., Davidson, A., Boots, M., and Buckling, A. (2015). Parasite exposure drives selective evolution of constitutive versus inducible defense. Curr. Biol. *25*, 1043–1049.

Wright, A.V., Nuñez, J.K., and Doudna, J.A. (2016). Biology and Applications of CRISPR Systems: Harnessing Nature's Toolbox for Genome Engineering. Cell *164*, 29–44.

Molecular Cell, Volume *64*

Supplemental Information

Quorum Sensing Controls Adaptive Immunity through the Regulation of Multiple CRISPR-Cas Systems

Adrian G. Patterson, Simon A. Jackson, Corinda Taylor, Gary B. Evans, George P.C. Salmond, Rita Przybilski, Raymond H.J. Staals, and Peter C. Fineran

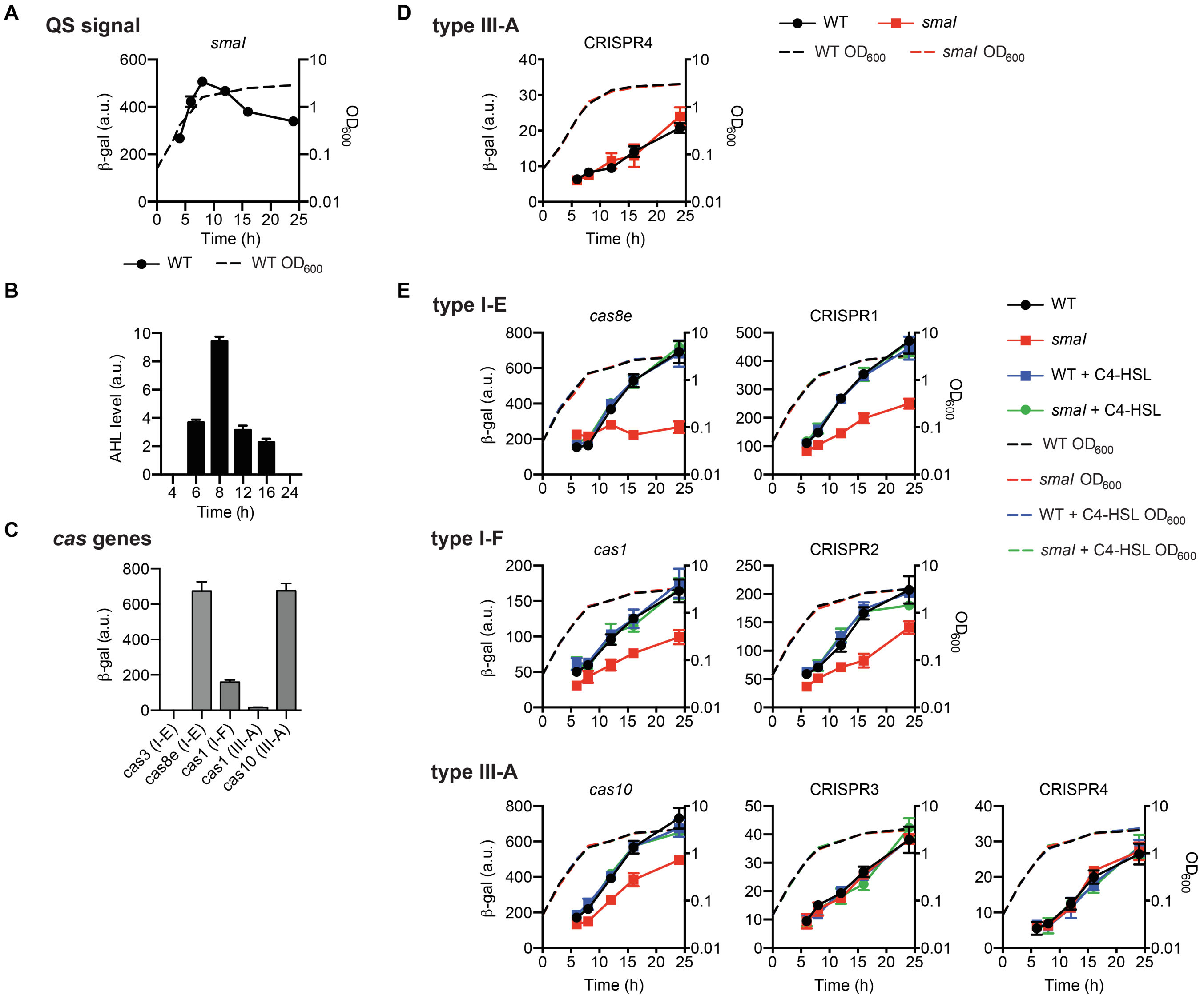

Figure S1. Production of C4-HSL signal via SmaI is responsible for upregulation of the I-E, I-F and III-A CRISPR-Cas systems at high cell density (related to Figure 1).

(A) Normalized *smaI*::*lacZ* reporter activity and cell growth of strain LC13. (B) AHL levels in the supernatant of WT cultures, measured using a bioassay. (C) Activity of the *cas*::*lacZ* reporters for the type I-E *cas3* (strain PCF209) and *cas8e* (strain PCF210), type I-F *cas1* (strain PCF211) and type III-A *cas1* (strain PCF212) and *cas10* (strain PCF213) promoters at 24 h post inoculation in the WT background. (D) Activity of CRISPR4::*lacZ* (solid lines) and growth (dashed lines) in the WT (strain PCF217) and *smaI* (strain PCF226) backgrounds. (E) Activity of *cas*::*lacZ* and CRISPR::*lacZ* for each of the type I-E, I-F and III-A CRISPR-Cas systems in the WT (strains PCF210, PCF211 and PCF213-PCF217) and *smaI* mutant backgrounds (PCF219, PCF220 and PCF222-PCF226) throughout growth ± 0.5 μM C4-HSL or DMSO (solvent control). Differences in activity between *smaI* + DMSO and *smaI* + C4-HSL beyond 12 h were statistically significant ($P \leq 0.05$) for each reporter except CRISPR3 and CRISPR4 (two-way analysis of variance (ANOVA) with the Bonferroni's multiple comparisons test). Data shown are the mean ± SD ($n = 3$).

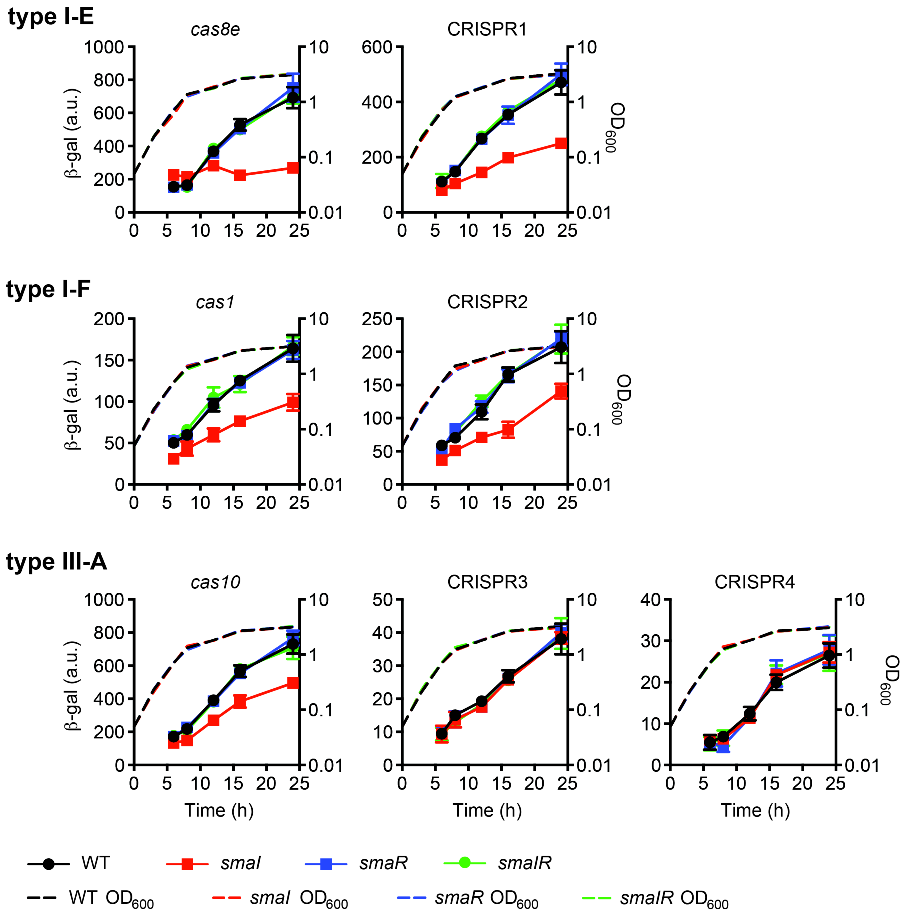

Figure S2. SmaR represses CRISPR-Cas expression in the absence of QS signals through growth (related to Figure 2). *cas*::*lacZ* and CRISPR::*lacZ* activity for each of the type I-E, type I-F and type III-A reporter strains in the WT (see Figure S1 for strain names), *smaI* mutant (see Figure S1 for strain names), *smaR* mutant (PCF228, PCF229 and PCF231-235) and *smaIR* mutant (PCF237, PCF238 and PCF240-244) backgrounds throughout growth. Differences in activity between *smaI* and all other strains beyond 12 h were statistically significant ($P \leq 0.05$) for each reporter except CRISPR3 and CRISPR4 (two-way analysis of variance (ANOVA) with the Bonferroni's multiple comparisons test). Data shown are the mean ± SD (n = 3).

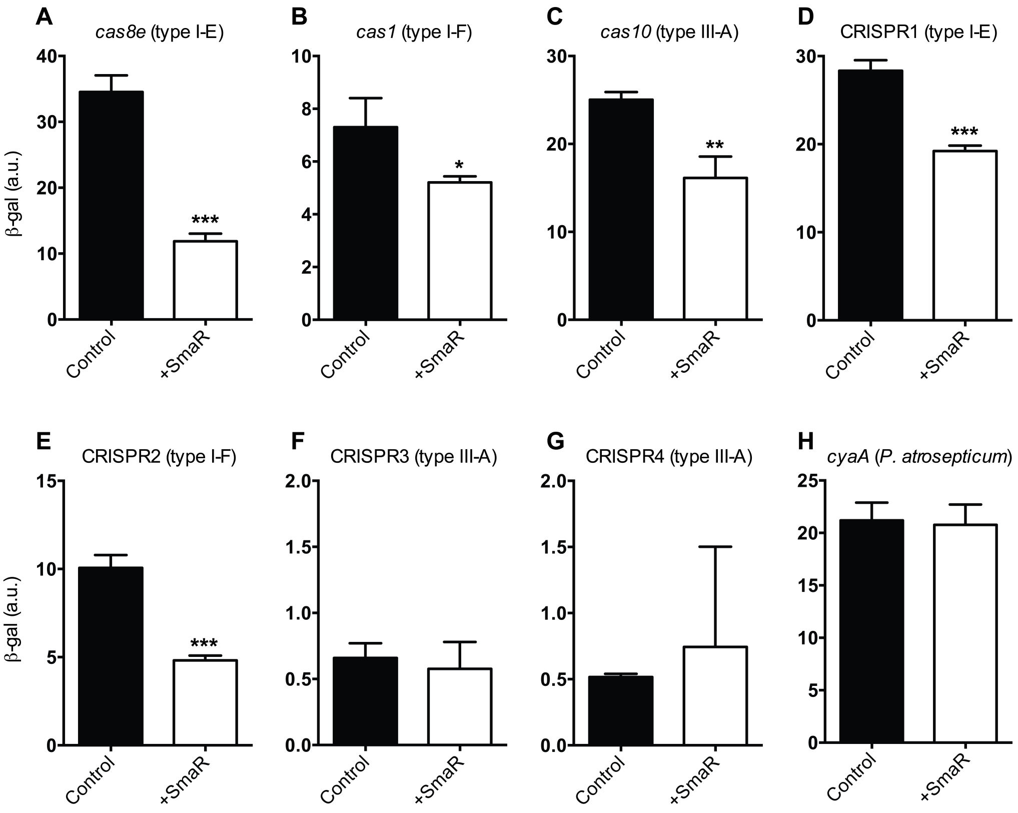

Figure S3. SmaR represses the type I-E, I-F and III-A CRISPR-Cas systems (related to Figure 2).

Normalized *cas*::*lacZ* activity in a heterologous system (*E. coli* CC118 λ*pir*) for each of the (A) type I-E *cas8e* (pPF714), (B) type I-F *cas1* (pPF715), (C) type III-A *cas10* (pPF717), (D) type I-E CRISPR1 (pPF861), (E) type I-F CRISPR2 (pPF862), (F) type III-A CRISPR3 (pPF863) and (G) type III-A CRISPR4 (pPF888) promoters ± SmaR (pPF972), at 3 h post induction with 0.1 mM IPTG. (H) Activity of a *P. atrosepticum cyaA*::*lacZ* (control) reporter in *E. coli* CC118 λ*pir* ± SmaR (pPF972), at 3 h post induction. Statistical significance was assessed by unpaired two-tailed t-test (* $P \leq 0.05$, ** $P \leq 0.01$, *** $P \leq 0.001$). Data shown are the mean ± SD ($n = 3$).

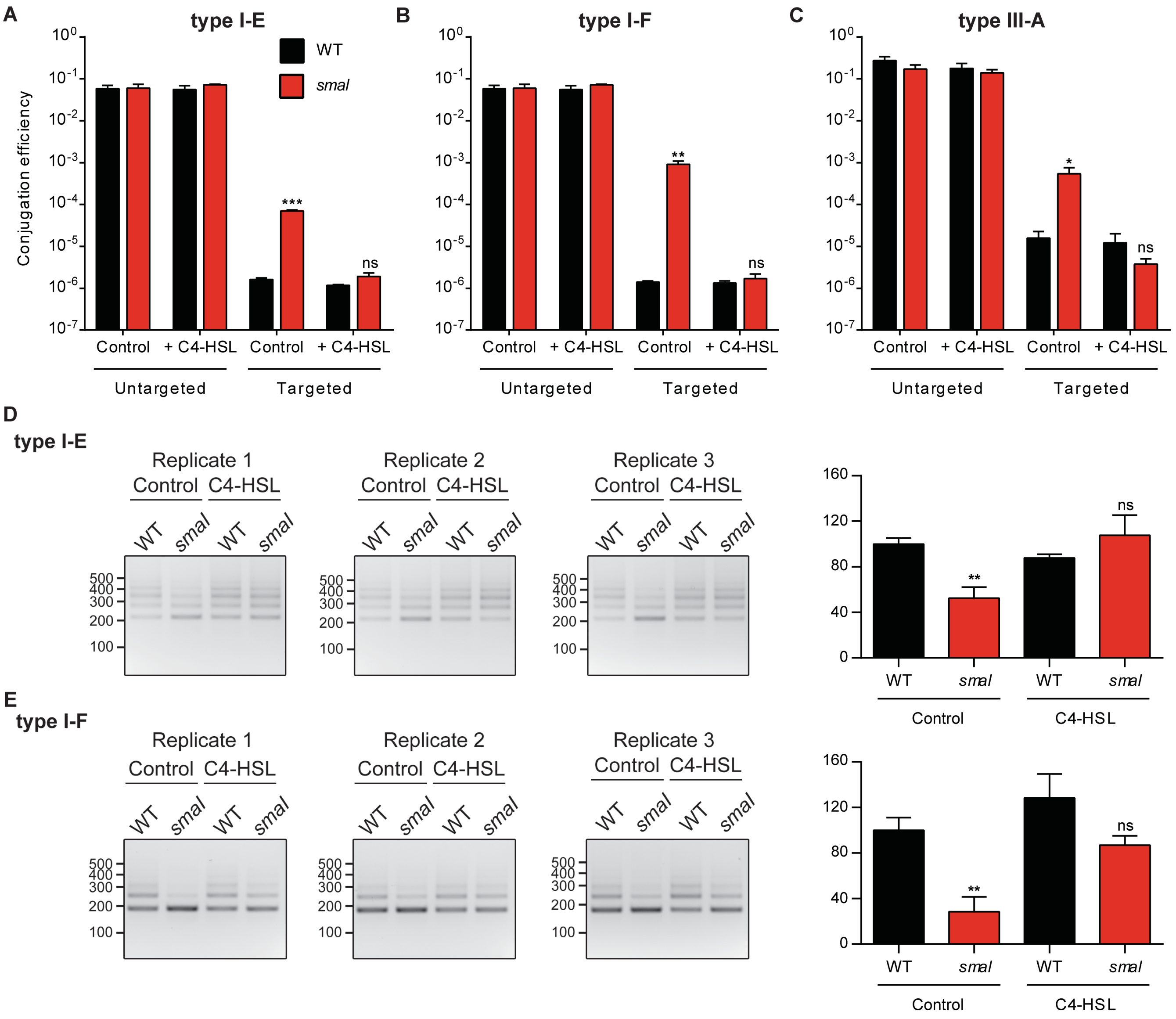

Figure S4. Addition of C4-HSL to the *smaI* mutant restores wild-type interference and adaptation for each CRISPR-Cas subtype (related to Figures 3 and 4).

Conjugation efficiency of untargeted control and targeted plasmids for the (A) type I-E (pPF719 control and pPF724 targeted), (B) type I-F (pPF719 control and pPF722 targeted) or (C) type III-A (pPF781 control and pPF1043 targeted) systems in WT (LacA) or *smaI* (LIS) mutant backgrounds. Conjugation efficiency was scored as transconjugants/recipients. Spacer acquisition (CRISPR expansion) for the WT or *smaI* mutant strains for either the (D) type I-E (pPF1048 primed) or (E) type I-F (pPF1032 primed) systems. CRISPR arrays were amplified by PCR and analyzed on 3% agarose gels. CRISPR expansion was normalized relative to the expansion observed in the WT control (WT control mean set as 100%). C4-HSL was added at a final concentration of 0.5 µM to the plates and bacterial cultures throughout the experiments and the controls included DMSO solvent. Statistical significance was assessed by unpaired t-test (* $P \leq 0.05$, ** $P \leq 0.01$, *** $P \leq 0.001$). Data shown are the mean ± SD ($n = 3$).

Table S1. Bacterial strains used in this study (related to Figures 1-4).

Name	Genotype/Phenotype	Reference
E. coli		
CC118 λ*pir*	*araD*, Δ(*ara*, *leu*), Δ*lacZ*74, *phoA*20, *galK*, *thi-1*, *rspE*, *rpoB*, *argE*, *recA*1, λpir	(Herrero et al., 1990)
DH5α	F$^-$, φ80Δd*lacZ*M15, Δ(*lacZYA–argF*)U169, *endA1*, *recA1*, *hsd*R17 ($r_K^-m_K^+$), *deoR*, *thi-1*, *sup*E44, λ$^-$, *gyr*A96, *rel*A1	Gibco/BRL
S17-1 λ*pir*	*recA, pro, hsdR, recA*::RP4-2-Tc::Mu, λ*pir,* Tmp^R, Sp^R, Sm^R	(de Lorenzo et al., 1990)
ST18	S17-1 λ*pir* Δ*hemA*	(Thoma and Schobert, 2009)
***Serratia* sp. ATCC 39006**		
LacA	lac- mutant generated by EMS mutagenesis, denoted "wild-type (WT)"	(Thomson et al., 2000)
ISTSO4	*smaI*::mini-Tn*5*Sm/Sp, *pigX*::mini-Tn*5lacZ1*, Sm^R/Sp^R, Km^R	(Fineran et al., 2005)
LC13	*smaI*::mini-Tn*5lacZ1*, Km^R	(Thomson et al., 2000)
LIS	*smaI*::mini-Tn*5*Sm/Sp, Sm^R/Sp^R	(Thomson et al., 2000)
MCR2000	*smaR*::*cat*, Cm^R	(Slater et al., 2003)
PCF208	*smaI*::mini-Tn*5*Sm/Sp, *smaR*::*cat*, Sm^R/Sp^R, Cm^R	This study
PCF209	WT type I-E *cas3*pro::*lacZ* (pPF713 integrant), Tc^R	This study
PCF210	WT type I-E *cas8e*pro::*lacZ* (pPF714 integrant), Tc^R	This study
PCF211	WT type I-F *cas1*pro::*lacZ* (pPF715 integrant), Tc^R	This study
PCF212	WT type III-A *cas1*pro::*lacZ* (pPF716 integrant), Tc^R	This study
PCF213	WT type III-A *cas10*pro::*lacZ* (pPF717 integrant), Tc^R	This study
PCF214	WT type I-E CRISPR1pro::*lacZ* (pPF861 integrant), Tc^R	This study
PCF215	WT type I-F CRISPR2pro::*lacZ* (pPF862 integrant), Tc^R	This study
PCF216	WT type III-A CRISPR3pro::*lacZ* (pPF863 integrant), Tc^R	This study
PCF217	WT type III-A CRISPR4pro::*lacZ* (pPF888 integrant), Tc^R	This study
PCF219	*smaI*::mini-Tn*5*Sm/Sp, type I-E *cas8e*pro::*lacZ* (pPF714 integrant), Tc^R, Sm^R/Sp^R	This study
PCF220	*smaI*::mini-Tn*5*Sm/Sp, type I-F *cas1*pro::*lacZ* (pPF715 integrant), Tc^R, Sm^R/Sp^R	This study
PCF222	*smaI*::mini-Tn*5*Sm/Sp, type III-A *cas10*pro::*lacZ* (pPF717 integrant), Tc^R, Sm^R/Sp^R	This study
PCF223	*smaI*::mini-Tn*5*Sm/Sp, type I-E CRISPR1pro::*lacZ* (pPF861 integrant), Tc^R, Sm^R/Sp^R	This study
PCF224	*smaI*::mini-Tn*5*Sm/Sp, type I-F CRISPR2pro::*lacZ* (pPF862 integrant), Tc^R, Sm^R/Sp^R	This study
PCF225	*smaI*::mini-Tn*5*Sm/Sp, type III-A CRISPR3pro::*lacZ* (pPF863 integrant), Tc^R, Sm^R/Sp^R	This study
PCF226	*smaI*::mini-Tn*5*Sm/Sp, type III-A CRISPR4pro::*lacZ* (pPF888 integrant), Tc^R, Sm^R/Sp^R	This study
PCF228	*smaR*::*cat*, type I-E *cas8e*pro::*lacZ* (pPF714 integrant), Tc^R, Cm^R	This study
PCF229	*smaR*::*cat*, type I-F *cas1*pro::*lacZ* (pPF715 integrant), Tc^R, Cm^R	This study
PCF231	*smaR*::*cat*, type III-A *cas10*pro::*lacZ* (pPF717 integrant), Tc^R, Cm^R	This study
PCF232	*smaR*::*cat*, type I-E CRISPR1pro::*lacZ* (pPF861 integrant), Tc^R, Cm^R	This study
PCF233	*smaR*::*cat*, type I-F CRISPR2pro::*lacZ* (pPF862 integrant), Tc^R, Cm^R	This study
PCF234	*smaR*::*cat*, type III-A CRISPR3pro::*lacZ* (pPF863 integrant), Tc^R, Cm^R	This study
PCF235	*smaR*::*cat*, type III-A CRISPR4pro::*lacZ* (pPF888 integrant), Tc^R, Cm^R	This study
PCF237	*smaI*::mini-Tn*5*Sm/Sp, *smaR*::*cat*, type I-E *cas8e*pro::*lacZ* (pPF714 integrant), Tc^R, Cm^R, Sm^R/Sp^R	This study
PCF238	*smaI*::mini-Tn*5*Sm/Sp, *smaR*::*cat*, type I-F *cas1*pro::*lacZ* (pPF715 integrant), Tc^R, Cm^R, Sm^R/Sp^R	This study
PCF240	*smaI*::mini-Tn*5*Sm/Sp, *smaR*::*cat*, type III-A *cas10*pro::*lacZ* (pPF717 integrant), Tc^R, Cm^R, Sm^R/Sp^R	This study
PCF241	*smaI*::mini-Tn*5*Sm/Sp, *smaR*::*cat*, type I-E CRISPR1pro::*lacZ* (pPF861 integrant), Tc^R, Cm^R, Sm^R/Sp^R	This study
PCF242	*smaI*::mini-Tn*5*Sm/Sp, *smaR*::*cat*, type I-F CRISPR2pro::*lacZ* (pPF862 integrant), Tc^R, Cm^R, Sm^R/Sp^R	This study
PCF243	*smaI*::mini-Tn*5*Sm/Sp, *smaR*::*cat*, type III-A CRISPR3pro::*lacZ* (pPF863 integrant), Tc^R, Cm^R, Sm^R/Sp^R	This study
PCF244	*smaI*::mini-Tn*5*Sm/Sp, *smaR*::*cat*, type III-A CRISPR4pro::*lacZ* (pPF888 integrant), Tc^R, Cm^R, Sm^R/Sp^R	This study

Table S2. Plasmids used in this study (related to Figures 1-4).

Name	Genotype/Phenotype	Reference
**Priming/interference**		
pBAD30	Arabinose inducible expression vector, Ap^R	(Guzman et al., 1995)
pQE-80LoriT-GFP	pQE-80LoriT derivative containing GFP, Ap^R	Josh Ramsay; unpublished
pQE-80LoriT-mCherry	pQE-80LoriT derivative containing mCherry, Ap^R	(Richter et al., 2014)
pPF719	Unprimed/naïve control pQE-80LoriT-GFP, Tc^R	This study
pPF1048	Primed type I-E CRISPR1 spacer 1 (TCA PAM) pQE-80LoriT-GFP-derivative, Tc^R	This study
pPF1032	Primed type I-F CRISPR2 spacer 2 containing a single nucleotide deletion at position 23 (GA PAM) pQE-80LoriT-GFP-derivative, Tc^R	This study
pPF724	Targeted type I-E CRISPR1 spacer 1 (CTT PAM) pQE-80LoriT-GFP-derivative, Tc^R	This study
pPF722	Targeted type I-F CRISPR2 spacer 1 (GG PAM) pQE-80LoriT-GFP-derivative, Tc^R	This study
pPF781	Untargeted/naïve control for the type III-A system, pBAD30 derivative, Cm^R	This study
pPF1043	Targeted type III-A pPF781 derivative containing a protospacer complementary to CRISPR3 spacer 1, Cm^R	This study
***Serratia* sp. ATCC 39006 *lacZ* reporters**		
pVIK107-Tc	pVIK107-derivative, integrative *lacZ* reporter plasmid, RP4 oriT, oriR6K, Tc^R	(Patterson et al., 2015)
pPF713	type I-E *cas3*pro::*lacZ* reporter, pVIK107-Tc-derivative, Tc^R	This study
pPF714	type I-E *cas8e*pro::*lacZ* reporter, pVIK107-Tc-derivative, Tc^R	This study
pPF715	type I-F *cas1*pro::*lacZ* reporter, pVIK107-Tc-derivative, Tc^R	This study
pPF716	type III-A *cas1*pro::*lacZ* reporter, pVIK107-Tc-derivative, Tc^R	This study
pPF717	type III-A *cas10*pro::*lacZ* reporter, pVIK107-Tc-derivative, Tc^R	This study
pPF861	type I-E CRISPR1pro::*lacZ* reporter, pVIK107-Tc-derivative, Tc^R	This study
pPF862	type I-F CRISPR2pro::*lacZ* reporter, pVIK107-Tc-derivative, Tc^R	This study
pPF863	type III-A CRISPR3pro::*lacZ* reporter, pVIK107-Tc-derivative, Tc^R	This study
pPF888	type III-A CRISPR4pro::*lacZ* reporter, pVIK107-Tc-derivative, Tc^R	This study
***Pectobacterium atrosepticum lacZ* reporters**		
pPF1008	*cyaA*::*lacZ* reporter, pVIK107-Tc-derivative, Tc^R	This study
**SmaR expression**		
pQE-80LoriT	pQE-80L (Qiagen) derivative containing RP4 oriT, Ap^R	(Richter et al., 2014)
pPF972	His_6-SmaR in pQE-80L-oriT, Ap^R	This study

Table S3. Oligonucleotides used in this study (related to Figures 1-4).

Name	Sequence (5'-3')	Notes	Restriction site(s)
PF209	TCGTCTTCACCTCGAGAAATC	F pQE-80L-oriT MCS	
PF210	GTCATTACTGGATCTATCAACAGG	R pQE-80L-oriT MCS	
PF565	CATAACACTGACAGAGGATCC	type I-E CRISPR1 sequencing	
PF575	GAACCCTTCAAAAATTGTCG	type I-F CRISPR2 sequencing	
PF610	CCTGGCAGTTCCCTACTCTC	R pBAD30 post-MCR	
PF633	GTGGATCTGGATGGACTGC	type I-E CRISPR1 sequencing	
PF1451	GCCACCTCGACCTGAAT	Targeted/primed construct sequencing	
PF1615	TTTTCCATGGGAATTGATTGGCTCCAATTC	F untargeted (no protospacer)	NcoI
PF1616	TTTTCCATGG**CN***CTGCAAAAATGCAGTAATATCAAGAAGTTTTAC* GAATTGATTGGCTCCAATTC	F type I-F CRISPR2 *protospacer* 1 (variable **PAM**)	NcoI
PF1617	TTTTCCATGG**ANG***TAAACAATTGCAGGACCAGCAAAATCTGTGG C*GAATTGATTGGCTCCAATTC	F type I-E CRISPR1 *protospacer* 1 (variable **PAM**)	NcoI
PF1623	TTTTCTAGAGGAATATAATTAACCTGATAATAAATG	F type I-E *cas3*pro	XbaI
PF1624	TTTCTGCAGCATCGCTTATTTTTAGAATAGAAC	R type I-E *cas3*pro	PstI
PF1625	TTTTCTAGATTTATCGGTGCGGGAAAGC	F type I-E *cas8e*pro	XbaI
PF1626	TTTCTGCAGCATGGATCTATCTCCTCAGTTA	R type I-E *cas8e*pro	PstI
PF1627	TTTTCTAGATCGTGTGCCAAACCCTTTTTTC	F type I-F *cas1*pro	XbaI
PF1628	TTTCTGCAGCATAATATATTCTCTGCTATCGCG	R type I-F *cas1*pro	PstI
PF1629	TTTTCTAGAATCCGCTCTCAGGGCTTTATG	F type III-A *cas1*pro	XbaI
PF1630	TTTCTGCAGCATGGTATCGATGTTCACTATGAAG	R type III-A *cas1*pro	PstI
PF1631	TTTTCTAGAAGTAGGTACTATTTCTTTGGTCC	F type III-A *cas10*pro	XbaI
PF1632	TTTCTGCAGCATTGACATCTCCTTGTGCC	R type III-A *cas10*pro	PstI
PF1642	CAGTCTAATTTGTTGACG	F screening type I-E *cas3*pro	
PF1643	GAGGTCGATATCTTTACC	F screening type I-E *cas8e*pro	
PF1644	CCATTTCTAAGCTGCCTG	F screening type I-F *cas1*pro	
PF1645	CCATGTTCTGATGCAGTC	F screening type III-A *cas1*pro	
PF1646	CATTACAGAATCATTGTCC	F screening type III-A *cas10*pro	
PF1666	CTTGACCCGGGCGCTAGCGGAGTGTATACTGGCTTACTATGTTG GCAC	F pBAD30 AraC	XmaI
PF1668	TTTTCTAGACTGGCAACAGATTGTGGAAC	F type I-E CRISPR1pro	XbaI
PF1669	TTTCTGCAGCATAGCTGTTTCCTTCTAAAAATATATACCTGTTTTA TAAGG	R type I-E CRISPR1pro	PstI
PF1670	TTTTCTAGAAAATCGTAGACTAATTATTTAATAGCG	F type I-F CRISPR2pro	XbaI
PF1671	TTTCTGCAGCATAGCTGTTTCCTAGTAACATTTCCACTTTAACGA TTTG	R type I-F CRISPR2pro	PstI
PF1672	TTTTCTAGACACACAACCGGCACACTC	F type III-A CRISPR3pro	XbaI
PF1673	TTTCTGCAGCATAGCTGTTTCCTAATGAAAATTTATAACCCATTG TTTTTATTTG	R type III-A CRISPR3pro	PstI
PF1674	TTTTCTAGACCCATTCAGGCACAGATTTTG	F type III-A CRISPR4pro	XbaI
PF1675	TTTCTGCAGCATAGCTGTTTCCTAATGAAAATTTATAACCCATTG TTTTTGTTTG	R type III-A CRISPR4pro	PstI
PF1698	CTCGGTACCATGGCATGCTGCAGTGGATGACCTTTTGAATGACC	F T4:Cm^R:T7 cassette	KpnI/NcoI/SphI /PstI
PF1699	GAGGGGTTTTTTGGGCCAGCCTCGCAGAGCAG	F OriT, overlaps PF1700	
PF1700	GAGGCTGGCCCAAAAAACCCCTCAAGACCCG	R T4:Cm^R:T7 cassette, overlaps PF1699	
PF1701	TTTGCCCGGGAGCGCTTTTCCGCTGC	R OriT	XmaI
PF1789	GATCCCGGGGTTTCAGGAAGGCGGCAAG	F primer *cyaA* pro	SmaI
PF1790	TTTTCTAGACAAGACGTTTCGCCTGATTTATC	R primer *cyaA* pro	XbaI
PF1834	GTTGGTGGTCGTGACGG	F screening type I-E CRISPR1pro and sequencing	
PF1835	GAATACGGCAGTGCTGGAG	F screening type I-F CRISPR2pro and sequencing	
PF1836	CCCAGAGGAAGACCGTC	F screening type III-A CRISPR3pro	
PF1837	CATCGTTCCATCATGCG	F screening type III-A CRISPR4pro	
PF1874	GGTCAATCAAGAATTTCACCAG	type I-E CRISPR1 sequencing	
PF1875	GGACAATACTTTTTGAGGATGAC	type I-E CRISPR1 sequencing	
PF1876	GTTTAGCTACTACTCCGCGCAGC	type I-F CRISPR2 sequencing	
PF1887	GTTAAGTCAGCAGGCGTTTAGTCG	R type I-E CRISPR1 spacer 2	

Table S3 (continued). Oligonucleotides used in this study (related to Figures 1-4).

Name	Sequence (5'-3')	Notes	Restriction site(s)
PF1888	CATCTGATGCTGACGACACTG	R type I-F CRISPR2 spacer 2	
PF1903	GTTGTTCCGCGAGACTATCGAC	type I-F CRISPR2 sequencing	
PF1904	CCAGGAATCACTATATCTGGCAAG	type I-F CRISPR2 sequencing	
PF1905	GCACTGAATGTTCGATATCATTAC	type I-E CRISPR1 sequencing	
PF1958	CTGGGATCCGTGTCTAATTCATTCTTTAAT	F *smaR*	BamHI
PF1959	GATCTGCAGTCATTCTGCGTCAGGGAG	R *smaR*	PstI
PF1989	TAAGTTAGTGTTCTTTAACAAGCAGGA	F type I-E CRISPR1 leader	
PF1990	CACGAAAATGATAATTGATGCTGAT	F type I-F CRISPR2 leader	
PF2034	TTTTCCATGG**N**C*TGGAAGCCGCCAGTGTCGTCAGATCAGATGT*GAATTGATTGGCTCCAATTC	F type I-F CRISPR2 *protospacer* 2 containing a single nucleotide deletion at position 23 (variable **PAM**)	NcoI
PF2085	TTTTCCATGG**NSW***TAAACAATTGCAGGACCAGCAAAATCTGTGGC*GAATTGATTGGCTCCAATTC	F type I-E CRISPR1 *protospacer* (variable **PAM**)	NcoI
PF2086	CTTGG*AAAAAAACCGACACGTAGTGTGAAAGAAATTAGG*ATGAGCATG	type III-A CRISPR3 *protospacer* 1, anneals to PF2087	KpnI/SphI compatible
PF2087	CTCAT*CCTAATTTCTTTCACACTACGTGTCGGTTTTTTT*CCAAGGTAC	type III-A CRISPR3 *protospacer* 1, anneals to PF2086	KpnI/SphI compatible

Note: In the oligonucleotide sequences, protospacers are in italics and restriction sites are underlined.

Supplemental Experimental Procedures.

CRISPR array sequencing

The *Serratia* sp. ATCC39006 genome has previously been sequenced (Fineran et al., 2013), but gaps existed at type I-E and I-F CRISPR arrays (CRISPR1 and CRISPR2). The CRISPR1 and CRISPR2 arrays were amplified by PCR from the LacA strain using primer pairs PF565 + PF1834 and PF565 + PF1835, respectively. The products were gel extracted and sequenced by primer walking using primers PF565, PF633, PF1874, PF1875 or PF1905 for CRISPR1 and PF575, PF1835, PF1876, PF1903 or PF1904 for CRISPR2. Assembled reads were mapped to the existing genome (NZ_AWXH00000000) using Geneious v9 (Kearse et al., 2012). The sequenced arrays were verified by aligning short (36 nt), single-end Illumina reads from the original de novo genome assembly dataset (Fineran et al., 2013). The CRISPR3 and CRISPR4 arrays were fully assembled in the existing draft sequence.

Construction of *cas* and CRISPR reporter strains

The *cas* and CRISPR promoter regions for the type I-E, I-F and III-A systems were each cloned into an integrative *lacZ* reporter plasmid, pVIK107-Tc (Table S2). The promoter regions were amplified by PCR and cloned into pVIK107-Tc using the primers and their respective restriction endonucleases listed in Table S3. The integrative vectors were introduced into the various *Serratia* backgrounds by conjugation from the donor strain *E. coli* S17-1 λpir. Counter-selection for the *E. coli* donor was achieved using either growth on minimal medium or on LB containing appropriate antibiotics for the *Serratia* backgrounds. All integrant reporter strains were confirmed by appropriate antibiotic resistance, PCR and sequencing.

Construction of SmaR expression plasmid and heterologous repression assays

A plasmid for heterologous expression of SmaR was generated by PCR amplification of the *smaR* gene using primers PF1958 and PF1959. The product was digested with BamHI and PstI and cloned into pQE-80LoriT that was cut with the same enzymes, resulting in plasmid pPF972. *E. coli* CC118 λ*pir* was transformed with either the vector control (pQE-80LoriT) or the construct for SmaR expression (pPF972) and with either the control reporter plasmid (pVIK107-Tc) or reporter plasmids for the different *cas* and CRISPR promoter-*lacZ* fusions (Table S2). Transformants were confirmed by PCR and their antibiotic-resistance profile, and grown at 37°C for β-galactosidase assays as described above.

Construction of interference and priming plasmids

Plasmids that would be targeted or primed by the type I-E and I-F systems were generated as follows. The gene encoding GFPmut3.1 was amplified from pGREENTIR and cloned into the EcoRI and HindIII sites of pQE-80LoriT, to generate pQE-80LoriT-GFP. The gene encoding Tc^R and either no protospacer (PF1615), a type I-E protospacer (PF1617) or a type I-F protospacer containing a single nucleotide deletion at position 23 (PF1616 and PF2034) was amplified from pTRB31 (Richter et al., 2014) with the reverse primer PF210. Primers PF1617, PF1616 and PF2034 had a variable nucleotide in the type I-E and I-F PAM, respectively, which allowed for cloning of targeted and primed plasmids (see Table S3). The PCR products were digested with NcoI and XhoI and ligated into pQE-80L-oriT-GFP cut with BspHI (compatible with NcoI) and XhoI. Plasmids were sequenced and the resulting plasmids were pPF719 (no protospacer control), pPF724 (type I-E targeted), pPF722 (type I-F targeted), pPF1048 (type I-E primed), pPF1032 (type I-F primed), (Table S3). To construct the type III-A targeted plasmid, we cloned a protospacer complementary to the CRISPR3 spacer 1 into the multiple cloning region a pBAD30 expression vector derivative (pPF781). The pPF781 (naïve control) plasmid was constructed by joining a Cm^R cassette with the RP4 OriT from pQE-80LoriT (using overlap PCR with primers PF1698 + PF1700 and PF1699 + PF1701 for the first round, followed by PF1698 + PF1701 in the second round), digesting the product with KpnI and XmaI, and subsequently ligating this with a KpnI/XmaI-digested AraC/P_{BAD} fragment of pBAD30 (generated by PCR using the primers PF1666 + PF610). The protospacer fragment corresponding to CRISPR3 spacer 1 was formed by annealing primers PF2086 and PF2087, which resulted in overlapping sticky ends compatible with the KpnI and SphI sites in the multiple cloning region of pPF781. The resulting construct (pPF1043) encodes the III-A targeted protospacer on the sense strand of the transcript produced from the *araBAD* promoter.

Supplemental References

Fineran, P.C., Slater, H., Everson, L., Hughes, K., and Salmond, G.P.C. (2005). Biosynthesis of tripyrrole and beta-lactam secondary metabolites in *Serratia*: integration of quorum sensing with multiple new regulatory components in the control of prodigiosin and carbapenem antibiotic production. Mol. Microbiol. *56*, 1495–1517.

Fineran, P.C., Iglesias Cans, M.C., Ramsay, J.P., Wilf, N.M., Cossyleon, D., McNeil, M.B., Williamson, N.R., Monson, R.E., Becher, S.A., Stanton, J.-A.L., et al. (2013). Draft Genome Sequence of *Serratia* sp. strain ATCC 39006, a Model Bacterium for Analysis of the Biosynthesis and Regulation of Prodigiosin, a Carbapenem, and Gas Vesicles. Genome Announc. *1*, e01039-13.

Guzman, L.L.M., Belin, D., Carson, M.J., Beckwith, J., Luz-Maria Guzman Michael J. Carson, and Jon Beckwith, D.B. (1995). Tight Regulation, Modulation, and High-Level Expression by Vectors Containing the Arabinose PBAD Promoter. J. Bacteriol. *177*, 4121–4130.

Herrero, M., De Lorenzo, V., and Timmis, K.N. (1990). Transposon vectors containing non-antibiotic resistance selection markers for cloning and stable chromosomal insertion of foreign genes in gram-negative bacteria. J. Bacteriol. *172*, 6557–6567.

Kearse, M., Moir, R., Wilson, A., Stones-Havas, S., Cheung, M., Sturrock, S., Buxton, S., Cooper, A., Markowitz, S., Duran, C., et al. (2012). Geneious Basic: An integrated and extendable desktop software platform for the organization and analysis of sequence data. Bioinformatics *28*, 1647–1649.

de Lorenzo, V., Herrero, M., Jakubzik, U., and Timmis, K.N. (1990). Mini-Tn5 transposon derivatives for insertion mutagenesis, promoter probing, and chromosomal insertion of cloned DNA in gram-negative eubacteria. J. Bacteriol. *172*, 6568–6572.

Patterson, A.G., Chang, J.T., Taylor, C., and Fineran, P.C. (2015). Regulation of the Type I-F CRISPR-Cas system by CRP-cAMP and GalM controls spacer acquisition and interference. Nucleic Acids Res. *43*, 6038–6048.

Richter, C., Dy, R.L., McKenzie, R.E., Watson, B.N.J., Taylor, C., Chang, J.T., McNeil, M.B., Staals, R.H.J., and Fineran, P.C. (2014). Priming in the Type I-F CRISPR-Cas system triggers strand-independent spacer acquisition, bi-directionally from the primed protospacer. Nucleic Acids Res. 8516–8526.

Slater, H., Crow, M., Everson, L., and Salmond, G.P.C. (2003). Phosphate availability regulates biosynthesis of two antibiotics, prodigiosin and carbapenem, in *Serratia* via both quorum-sensing-dependent and -independent pathways. Mol. Microbiol. *47*, 303–320.

Thoma, S., and Schobert, M. (2009). An improved *Escherichia coli* donor strain for diparental mating. FEMS Microbiol. Lett. *294*, 127–132.

Thomson, N.R., Crow, M. a., McGowan, S.J., Cox, A., and Salmond, G.P.C. (2000). Biosynthesis of carbapenem antibiotic and prodigiosin pigment in *Serratia* is under quorum sensing control. Mol. Microbiol. *36*, 539–556.
